# Submolecular‐Resolution Probing of Vibrational Anharmonicity Using Tip‐Enhanced Raman Spectroscopy

**DOI:** 10.1002/anie.202514215

**Published:** 2025-09-25

**Authors:** Youngwook Park, Ikutaro Hamada, Martin Wolf, Akitoshi Shiotari

**Affiliations:** ^1^ Department of Physical Chemistry Fritz‐Haber Institute of the Max‐Planck Society Berlin Germany; ^2^ Department of Precision Engineering Graduate School of Engineering The University of Osaka Suita Japan

**Keywords:** Anharmonic coupling, Combination bands, Overtones, Submolecular resolution, TERS

## Abstract

Vibrational spectroscopy can reach atomic spatial resolution via highly confined optical probes, offering local chemical insight. Yet, overtones and combination bands have not fully benefited due to weak transition moments. Here, we show submolecular‐resolution detection of intense overtones and combination bands using tip‐enhanced Raman spectroscopy (TERS) for an asymmetric perylene derivative on silicon. The Raman peaks, barely visible in tunneling regime, are ∼10 times enhanced when the tip‐apex contacts the molecule. This point‐contact‐mode TERS enables analysis of two anharmonicities with submolecular contrast: one arising from deviations of potential energy surfaces from harmonic shape (mechanical anharmonicity), the other from high‐order polarizability derivatives (electrical anharmonicity). Spatial variation of mechanical anharmonicity reveals a vibrational energy exchange channel at the submolecular scale, highlighting potential to map energy transfer in real space.

## Graphical abstract

This work explores how tip‐enhanced Raman spectroscopy achieves submolecular resolution in detecting vibrational overtones and combination bands. This breakthrough reveals site‐specific anharmonicities and energy transfer pathways within single molecules, advancing chemical analysis with unprecedented spatial and spectral detail.

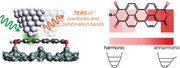

## Introduction

Vibrational anharmonicity is a fundamental concept for understanding the shape of potential energy surfaces (PES),^[^ ^1^, ^2^, ^3^ ^]^ bond‐selective chemistry,^[^ ^4^, ^5^, ^6^ ^]^ and vibrational energy exchange between intra‐ or intermolecular modes.^[^ ^7^, ^8^, ^9^, ^10^, ^11^, ^12^ ^]^ It manifests spectroscopically as overtone and combination band transitions associated with fundamental vibrational modes. Spectroscopic detection of anharmonicity, in either frequency^[^ ^13^, ^14^ ^]^ or time domain,^[^ ^15^, ^16^, ^17^, ^18^, ^19^, ^20^, ^21^, ^22^ ^]^ thus provides critical insights into chemical and physical processes such as bond breaking induced by overtone excitation,^[^ ^23^, ^24^, ^25^, ^26^ ^]^ vibrational energy relaxation via anharmonic mode coupling within molecules^[^ ^11^ ^]^ or with surfaces,^[^ ^8^, ^10^, ^12^, ^27^, ^28^ ^]^ as well as into the dynamics of energy flow in systems with highly anharmonic bonds.^[^ ^17^, ^21^, ^22^, ^29^ ^]^ Additionally, the detection of overtones and combination bands aids the reliable assignment of fundamental vibrational transitions. Nevertheless, spatially averaging spectroscopies are often complicated by anisotropic and heterogeneous molecular environments in the sample, as anharmonicity is highly sensitive to molecular conformation and the local environment.^[^ ^30^, ^31^, ^32^, ^33^ ^]^ These complexities hinder straightforward interpretation of spectra from molecular ensembles.

Single‐molecule detection of vibrational anharmonicity via local probes offers a solution to these challenges. For example, analysis of electron‐induced single‐molecule dynamics on surfaces by scanning tunneling microscopy (STM), namely action spectroscopy, has revealed the excitation of vibrational overtones mediating molecular diffusion and desorption.^[^ ^9^, ^34^, ^35^, ^36^ ^]^ Also in STM‐based inelastic electron tunneling spectroscopy (IETS), overtone transitions of single carbon monoxide molecules have been detected together with their energy shifts depending on the adsorption environment.^[^ ^37^ ^]^ However, both methods have so far been applied only to characterize overtones of small, mainly diatomic, molecules adsorbed on metals. For larger polyatomic molecules, submolecular spatial resolution would be essential, as local variations in the PES governing atom motion and vibrational coupling can occur within a single molecule. Furthermore, the localization of vibrational modes is more pronounced at higher levels of vibrational excitations than in the fundamentals, as anharmonicity weakens the coupling of identical oscillators, making the vibrational eigenstates resemble primarily the excitation of localized vibrations.^[^ ^38^, ^39^, ^40^, ^41^ ^]^

Tip‐enhanced Raman spectroscopy (TERS) based on STM under low‐temperature and ultrahigh‐vacuum (UHV) conditions is a powerful tool for probing vibrations of polyatomic molecules with atomic‐scale spatial resolution.^[^ ^42^, ^43^, ^44^, ^45^ ^]^ However, the detection of overtone and combination band transitions using TERS has been scarce and largely restricted to preliminary observations without systematic investigation with submolecular spatial resolution.^[^ ^46^, ^47^, ^48^ ^]^ Even in conventional Raman spectroscopy of molecular ensembles, these transitions are often too weak to detect due to selection rules, requiring electronic resonance with excitation laser wavelength.^[^ ^49^, ^50^, ^51^, ^52^ ^]^ Although surface‐enhanced Raman spectroscopy (SERS) has successfully enabled single‐molecule detection of overtone and combination transitions via electronic resonance,^[^ ^53^, ^54^, ^55^, ^56^, ^57^, ^58^ ^]^ the complexity and influence of the local environment imply challenges for precise spatial characterization, motivating the use of local probes like TERS to achieve submolecular resolution under well‐defined conditions.

**Figure 1:**
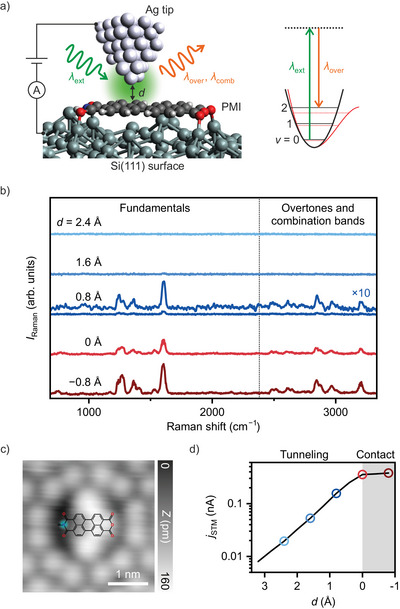
Detection of overtones and combination bands with point‐contact TERS. a) Schematic of TERS based on STM, where *d* denotes the tip–molecule distance. The right panel shows the schematic of Raman process for a vibrational overtone (*v*: vibrational quantum number). b) TERS spectra of PMI/Si(111) recorded at five different *d*. The spectra are vertically offset for clarity. The spectrum at *d* = 0.8 Å is displayed alongside its ×10 magnification. c) Constant‐current STM image of PMI/Si(111), acquired with the sample bias *V*_bias_ of 0.8 V and the STM current *j*_STM_ of 50 pA. The molecular structure of PMI is overlaid on the image. The cyan circle marks the tip position used for TERS in (b) and STM current measurements in (d). d) STM current measured as a function of *d*. Circles in the plot indicate the positions where the spectra in (b) were acquired. The gray‐shaded area represents the contact regime (*d* ⩽ 0).

In this study, we employed STM‐based TERS at low temperature in the point‐contact mode^[^ ^59^ ^]^ to reliably measure vibrational anharmonicity in single molecules adsorbed on a silicon surface (Figure 1a). Pronounced overtones and combination bands were observed for an asymmetric perylene derivative, revealing submolecular spatial variations in vibrational anharmonicity. Spatial contrasts were identified in both mechanical (deviation from harmonic potential) and electrical (high‐order polarizability derivatives) components of anharmonicity at a submolecular level, and their origins are explored.

## Results and Discussion

### Enhancement of Overtone and Combination Band Intensities in Point‐Contact‐Mode TERS

Point‐contact formation between an STM tip and a molecule can significantly enhance TERS intensity^[^ ^48^, ^59^, ^60^, ^61^, ^62^, ^63^ ^]^ due to the tip–molecule interaction. This enhancement facilitates the robust detection of weak overtone and combination transitions from single molecules. Figure 1b presents a series of TERS spectra of perylene‐3,4,9,10‐tetracarboxylic monoimide monoanhydride (PMI) adsorbed on a Si(111)‐7×7 reconstructed surface with varying tip–molecule distance (*d*), showing the impact of point‐contact formation on the intensity of these transitions. The Si(111)‐7×7 provides a stable adsorption site for PMI at a corner hole, by forming strong O–Si bonds with the four carbonyl groups of PMI. Importantly, the asymmetric nature of PMI is clearly visible in its STM image, where the imide side appears as a depression and the anhydride side as a protrusion (Figure 1c).^[^ ^62^ ^]^ Therefore, PMI/Si(111) serves as an ideal system for this study, not only due to the well‐characterized geometry and properties under light illumination,^[^ ^62^ ^]^ but also due to the possible localization of electronic states and vibrations. Notably, Si is nonplasmonic in the visible range, making TERS acquisition inherently challenging on such substrates.^[^ ^48^, ^62^, ^64^ ^]^

To obtain the spectra in Figure 1b, the tip was first positioned laterally above the imide group of PMI in the tunneling regime (tip–molecule distance *d* ≈ 5 Å), as illustrated in the STM image (Figure 1c), and then vertically adjusted (see Methods for details on tip positioning procedure). As shown in the tip‐height dependence of STM current (Figure 1d), the current saturation defines the point of contact formation *d* = 0,^[^ ^48^, ^59^, ^62^ ^]^ distinguishing the contact regime (*d* ⩽ 0) from the tunneling regime (*d* > 0). A sharp increase in TERS intensity (∼10 times) is observed for *d* ⩽ 0, making overtones and combination bands clearly visible. Notably, these transitions appear even before contact formation in the tunneling regime (*d* = 0.8 Å in Figure 1b), with spectral shapes nearly identical to those in the contact regime (e.g., *d* = −0.8 Å in Figure 1b). This indicates that tip–molecule contact does not induce anharmonicity but instead just enhances signal intensity. Detailed signal intensity analysis and subtle spectral changes associated with contact formation are discussed in Section S2.

### Overtone and Combination Band Assignments in TERS Spectra

**Figure 2:**
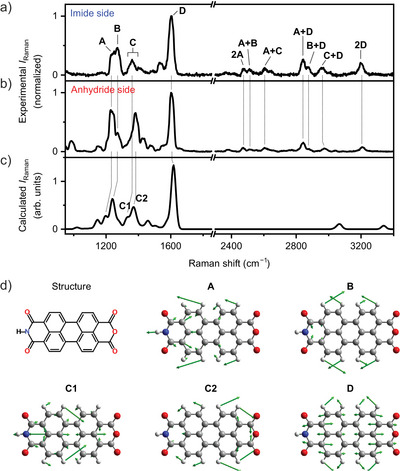
Spectral assignment of overtones and combination bands in PMI/Si(111) TERS spectrum. a,b) Experimental TERS spectrum of PMI/Si(111) acquired at two different tip positions, on the N atom of the imide group a) and on the central O atom of the anhydride group b). *d* = −0.3 Å. Each spectrum is normalized to the intensity of its peak **D**. c) Calculated Raman spectrum of PMI. d) Molecular structure and calculated atomic displacements for the normal modes **A**–**D** of PMI.

Figure 2a presents the point‐contact TERS spectrum of PMI/Si(111) acquired with the tip positioned at the imide N atom. Despite using a different tip and probing a different individual PMI/Si(111) molecule than in Figure 1b, the resulting spectrum remained nearly identical, demonstrating the reproducibility of the measurement. We focus primarily on four vibrational modes, arbitrarily labeled **A**–**D**, at 1236.9, 1270.7, 1359.9, and 1602.7 cm^−1^, respectively, which are the strongest fundamental transitions in the spectrum. We also observed prominent peaks in the energy range of 2470–3210 cm^−1^, which are assigned to the first overtone and combination transitions of modes **A**–**D**, as labeled in Figure 2a, based on peak positions and intensities (Table S1). Note that the C–H stretching fundamental vibrations of perylene, which typically appear slightly above 3000 cm^−1^, may overlap with the overtone and combination bands of **A**–**D**. However, the contribution of these C–H stretching modes to the overall spectrum is minimal, as inferred from the peak assignment (see Figure S1). The absence of visible C–H stretching in the spectrum suggests that Fermi resonance with the overtones and combinations is unlikely.

Figure 2b presents the TERS spectrum of the same PMI molecule recorded with the tip fixed above the central O atom of the anhydride group. We assigned the fundamental peaks **A**–**D** based on the similarity in Raman shift to those observed in Figure 2a. Despite some spectral differences, particularly in the relative intensity of peak **C**, the spectrum still displays pronounced overtones and combination bands, which are assigned similarly to those observed above the imide N atom (see Table S2).

The experimental fundamental peaks are reproduced by density functional theory (DFT) calculations, as shown in Figure 2c. To account for the distinct spectral features of the PMI/Si(111) system compared to those of the molecular powder (Figure S2) or an isolated molecule, we employed a PMI geometry optimized with the Si surface included, and subsequently computed vibrational properties using the molecular structure alone (see Methods section for details). The normal modes **A**–**D** correspond to collective in‐plane motions of atoms, primarily within the perylene structure, as illustrated by the atomic displacements in Figure 2d. Strong TERS intensities arise from these in‐plane vibrations due to the enhanced sensitivity to in‐plane polarizability changes at close tip–sample proximity.^[^ ^65^ ^]^ We highlight that the experimental peak **C** likely corresponds to two closely spaced calculated modes, labeled **C1** and **C2**. As shown in Figure 2d, mode **C1** involves atomic motions predominantly localized on the imide side, whereas mode **C2** is more confined to the anhydride side. Given that peak **C** in Figure 2b shows significantly higher relative intensity than in Figure 2a, we infer that the peak recorded at the imide site reflects a stronger contribution from mode **C1**, while the peak at the anhydride site corresponds primarily to mode **C2**. This assignment is further supported by the observation that peak **C** exhibits the largest Raman shift variation among all detected modes, shifting from 1359.9 cm^−1^ at the imide site to 1380.4 cm^−1^ at the anhydride site (20.5 cm^−1^ difference; Tables S1 and S2).

As a technical note on measuring overtones and combination bands using TERS, we investigated how their intensity depends on the nanoscale tip‐apex shape and incident laser wavelength. The results confirm that these transitions are enhanced by resonances with the localized surface plasmon of the junction (see Section S3).

### Submolecular Variation in Vibrational Anharmonicity: Mechanical and Electrical Contributions

The pronounced appearance of overtones and combination bands in Raman spectra reflects substantial vibrational anharmonicity of the system. This anharmonicity arises from two primary contributions:^[^ ^66^ ^]^ 1) mechanical anharmonicity, which refers to deviations of the PES from the harmonic approximation, and 2) electrical anharmonicity (also known as electronic anharmonicity), which arises from second‐order or higher derivatives of polarizability along vibrational coordinates (see detailed expressions in Section S4). Mechanical anharmonicity manifests in the Raman spectra as a downshift in the overtone (see the schematic energy curves on the right side of Figure 1a; black for harmonic and red for anharmonic) or combination band frequency with respect to the sum of the fundamental frequencies, defined as Δ_*i* + *j*_ = ν_*i* + *j*_ − (ν_*i*_ + ν_*j*_) where *i*, *j* = **A**, **B**, **C**, and **D** and ν_*i*_ denotes the measured Raman shift of mode *i*. The intensity of an overtone or combination transition relative to its corresponding fundamental(s) is influenced by both mechanical and electrical anharmonicities. Therefore, when a transition is mechanically harmonic, i.e., Δ_*i* + *j*_ = 0, electrical anharmonicity can be effectively monitored by its intensity.

**Figure 3:**
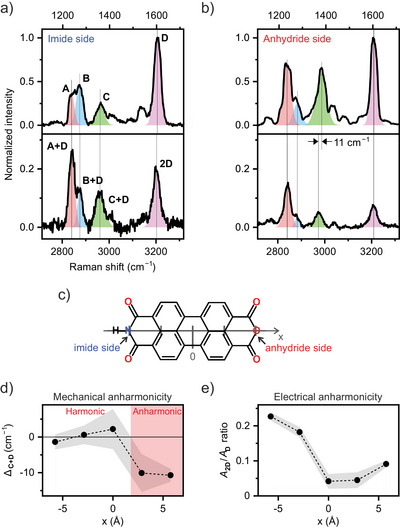
Spatially resolved vibrational anharmonicity of PMI/Si(111). a,b) TERS spectra of the fundamentals (top) and the first overtone and combination transitions (bottom) at two different tip positions, displayed for the comparison of vibrational anharmonicity of the combinations with mode **D**. The spectra are identical to those in Figure 2a,b. Four fundamental transitions **A**–**D** and their corresponding combinations with **D** (that is, **A+D**, **B+D**, **C+D**, and **2D**) are shown with same colors. Vertical lines indicate the position of the peaks of interest. c) Definition of the *x* axis for the line profiles in (d) and (e), with tick marks indicating the lateral tip positions where anharmonicities were examined. d) Line profile of Δ_**C + D**_ across the molecule. The red shading area serves as a guide for the positions where Δ_**C + D**_ < 0 beyond the error margin. e) Line profile of *A*_**2D**_/*A*_**D**_ ratio. Grey shading ribbons in (d) and (e) represent the vertical errors of the points based on the peak fitting.

TERS spectra acquired at different locations on a single molecule enable investigation of mechanical and electrical anharmonicities with submolecular spatial resolution. In Figure 3, we compare the fundamentals **A**–**D** and their corresponding combinations with mode **D** for two different tip positions on PMI: at the imide site in Figure 3a and the anhydride site in Figure 3b. The spectra in Figure 3a,b are identical to those shown in Figure 2a,b respectively, but are presented differently to more effectively visualize the two types of vibrational anharmonicity.

Two distinct types of site‐dependence in vibrational anharmonicity were identified from the spectra. The first type, mechanical anharmonicity, is examined by considering the downshift Δ_*i* + *j*_ for several *i* with *j* fixed to **D** (Figure 3a,b). Although other overtones and combinations show negligible downshift, the **C+D** combination measured at the anhydride site exhibits a notable shift, Δ_**C + D**_ = −11 cm^−1^, as indicated by the vertical offset lines in Figure 3b. This observed downshift implies pronounced anharmonic coupling between modes **C** and **D**. Intriguingly, this anharmonic coupling changes across the PMI molecule. Figure 3d presents a line profile of Δ_**C + D**_ across PMI (see also Figure S3a for a heatmap). Although the **C+D** combination mode is nearly harmonic at the imide side and at the center of the molecule, it becomes anharmonic at the anhydride side with negative Δ_**C + D**_ values exceeding the error margins. Such a submolecular variation in anharmonicity has not been previously reported by any experimental approach. The spatial contrast in the **C+D** anharmonic downshift was consistently reproduced across measurements employing varying tips and samples (Figure S4).

The second type of submolecular contrast appears in electrical anharmonicity. Figure 3e presents a line profile of the intensity of the overtone transition of mode **D**, *A*_**2D**_, relative to that of the fundamental, *A*_**D**_ (see Figure S3b for a heatmap). The *A*_**2D**_/*A*_**D**_ ratio is larger when the tip is positioned closer to the imide or anhydride group compared to the center of the molecule. Additionally, the *A*_**2D**_/*A*_**D**_ ratio is higher on the imide side than on the anhydride. It should be noted that the relative overtone intensity does not directly correlate with the absolute intensity of the fundamental modes. For instance, while the *A*_**2D**_/*A*_**D**_ ratio is higher at the imide group, the absolute intensity of mode **D** (*A*_**D**_) is considerably larger at the anhydride group (see Figure S5). Since **2D** is mechanically harmonic at all sites (i.e., Δ_**2D**_ = 0), the site variation of the *A*_**2D**_/*A*_**D**_ ratio must originate from the electrical component, reflecting a site‐dependent second‐order polarizability derivative along mode **D**. Based on this site dependence in PMI, we presume that the pronounced electrical anharmonicity in our systems is largely amplified by the presence of the O–Si bonds with the substrate (see Section S5 for details). The second‐order derivatives of polarizability are mode‐specific; for example, in Figure 3a, the combination **A+D** is approximately twice as intense as **B+D**, while the fundamentals **A** and **B** have comparable intensities. This suggests that the polarizability change along **A** with mode **D** is larger than along **B**.

**Figure 4:**
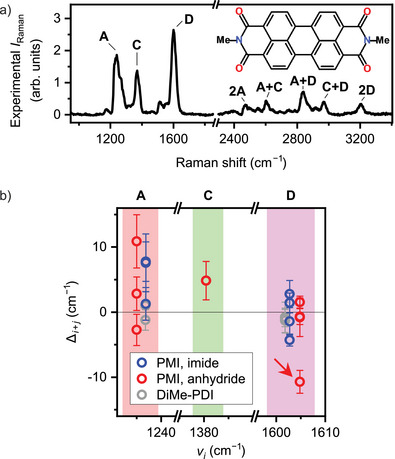
a) Experimental TERS spectrum of DiMe‐PDI/Si(111) acquired at the N atom of the imide group. *d* = −0.8 Å. b) Compilation of Δ_*i* + *j*_ for PMI and DiMe‐PDI plotted against fundamental peak positions. The red arrow indicates the data point corresponding to the **C+D** combination of PMI anhydride site.

As a structurally symmetric reference, we examine in Figure 4a TERS spectrum of perylene derivative, N,N'‐dimethyl perylene‐3,4,9,10‐tetracarboxylic diimide (DiMe‐PDI) on the Si(111)‐7×7 corner hole. The symmetric adsorption structure is confirmed by the STM image and the DFT optimization (Figure S6). Strong overtone and combination transitions for modes **A**–**D**, assigned in a manner similar to PMI, were also observed for DiMe‐PDI. Detailed assignments and calculated atomic motions of normal modes for DiMe‐PDI can be found in SI (Table S3, Figures S7 and S8) In contrast to PMI, DiMe‐PDI exhibits symmetry in its vibrations for the peak **C** (Figure S7c).

### Evaluation of Mechanical Anharmonicity

Beyond the combinations with mode **D** of PMI (Figure 3), we analyzed Δ_*i* + *j*_ values for all observed overtone and combination bands of PMI and DiMe‐PDI, which are summarized in the collage in Figure 4b. Nearly all the data points are distributed around Δ_*i* + *j*_ = 0, indicating the mechanically harmonic transitions. However, one notable outlier exhibits a downshift due to mechanical anharmonicity: the **C+D** combination for PMI at the anhydride site (Figure 3b), marked by an arrow in Figure 4b. Note that the deviations with Δ_*i* + *j*_ > 0 obtained for some transitions in Figure 4b are attributed to errors stemming from the assumption of a Gaussian peak shape in the peak‐fitting process. These pronounced positive deviations are primarily associated with **A** and **B** (Tables S1 and S2), suggesting that the peak decomposition introduced errors in the peak position.

**Figure 5:**
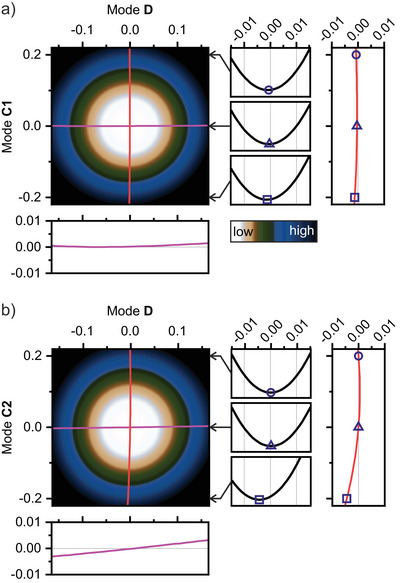
Calculated 2D PES for modes **C1** a) and **C2** b) with mode **D** of PMI, highlighting features of anharmonic coupling. The axes represent dimensionless normal mode displacements. The nearly vertical red lines indicate the energy‐minimum coordinates of mode **D** at each displacement along modes **C1** and **C2**, respectively. The 1D potential curves on the right side of each 2D PES, marked with these energy mimina, illustrate how the red lines are obtained. Similarly, the nearly horizontal magenta lines mark the energy‐minimum coordinates of modes **C1** and **C2** at each displacement along mode **D**. Both red and magenta lines are shown magnified in separate plots for visual clarity. The color scale in both 2D PES plots spans from 0 to 3(ν_C1, 2_ + ν_D_).

Based on the normal mode analysis of the PMI molecule, we constructed two‐dimensional (2D) PES for modes **C1** (Figure 5a) and **C2** (Figure 5b) in combination with mode **D**. This allows for the evaluation of both the mechanical anharmonicity of individual modes and the anharmonic coupling between them.^[^ ^1^ ^]^ The nearly symmetric shapes of the 2D PES in Figure 5 indicate predominantly harmonic behavior of each vibration (see Figure S9 for comparison with anharmonic oscillators), which agrees well with the harmonic overtones observed experimentally.

To identify subtle differences in anharmonic coupling, we traced the minimum‐energy coordinates along one mode as a function of displacement along the other. A comparison of Figure 5a,b reveals that mode **D** exhibits a more pronounced nonlinear shift in the minimum‐energy path when coupled with mode **C2** than with **C1**, suggesting stronger anharmonic coupling between **D** and **C2**. Considering our assignment of the experimental peak **C** at the imide site primarily to mode **C1**, and at the anhydride site to **C2**, this contrast in anharmonic coupling aligns well with the observed spatial variation in the mechanical anharmonicity of the **C+D** combination band.

Our findings suggest that the intrinsic asymmetry of PMI substantially contributes to the submolecular contrast observed in the mechanical anharmonicity of the **C+D** combination. First, regardless of the adsorption configuration, mechanical anharmonicity was consistently observed only at the anhydride side when monitoring the **C+D** combination band. By positioning the tip within a specific *d* range of 1–2 Å on the anhydride side of PMI under light irradiation,^[^ ^62^ ^]^ or in rare events when the tip was placed close on the imide group and then retracted, we could record spectra of PMI in different adsorption configurations: partially picked up at the anhydride side or fully picked up at the imide group by the tip (Figure S10). In these spectra, anharmonic mixing of **C** and **D** was observed exclusively at the anhydride side. This consistency across various molecular configurations suggests that the effect is intrinsic to the molecular structure rather than being induced by external factors. The observed nearly doubled downshift of the **C+D** band in the partially lifted PMI (∼−20 cm^−1^; see Figure S10) indicates that the substrate may impose additional asymmetry via non‐symmetric adsorption geometry. Second, the **C+D** combination band of the structurally symmetric DiMe‐PDI remains mechanically harmonic both in its flat adsorbed configurations (Figure 4b) and when partially picked up (Figure S11). It is noteworthy that previous SERS studies of symmetric perylene vibrations have similarly reported minimal mechanical anharmonicity.^[^ ^54^, ^57^ ^]^ The absence of mechanical anharmonicity in DiMe‐PDI further supports the idea that molecular asymmetry in PMI is the key factor driving the observed submolecular contrast.

As the tip remained in contact with the molecule during spectral acquisition, we cannot entirely exclude the possible influence of tip–molecule interactions, such as charge transfer, structural deformation, or plasmonic effects, on the observed mechanical anharmonicity. However, based on our observations, the tip influence does not appear to be a dominant factor in the mechanical anharmonicity of the system studied. Although the tip–imide and tip–anhydride interactions may differ, the tip–perylene interaction should be essentially identical on both sides of the molecule, which cannot account for the significant difference in Δ_**C + D**_ observed between the second and fourth data points in Figure 3d. Furthermore, tip–anhydride contact did not induce an anharmonic downshift in a symmetric perylene‐3,4,9,10‐tetracarboxylic dianhydride molecule (Figure S11), suggesting that the intrinsic molecular asymmetry of PMI is a critical factor in the observed mechanical anharmonicity. In addition, two positions on the anhydride side of PMI that yielded similar Δ_**C + D**_ values likely involve different tip–molecule interactions, one primarily involving the anhydride group and the other the perylene core. This further suggests that local tip–molecule interaction alone cannot explain the observed asymmetry in the mechanical anharmonicity. The subtle distinction between the 2D PES in Figure 5 was reproduced without including the tip in the calculations, supporting this interpretation.

The anharmonic coupling between two vibrations signifies the presence of a vibrational energy exchange channel between them. When two modes are coupled through an anharmonic 2D PES, their motions become mixed, more pronounced at higher combinational excitations, leading to behavior that deviates from a linear sum of their fundamentals. This mixing is thought to underlie the mechanism of vibrational energy transfer. The deviation from the fundamentals can be better understood by analogy to overtones, vibrational motion of which differs from the fundamental due to the influence of an anharmonic potential.

Spatially resolved mechanical anharmonicity thus may offer a promising means to probe vibrational energy flow within a molecule. The spatial variation of mechanical anharmonicity observed in PMI (Figure 3d) could suggest asymmetric intramolecular energy redistribution. To further explore the potential of this approach, spatially resolved detection of anharmonic downshift in higher‐order combination bands, beyond the first combinations discussed in this work, is desirable for a more definitive characterization of vibrational energy exchange pathways. Although second overtones and combination bands were observed under certain conditions in our measurements (see Figure S12), challenges remain, particularly regarding signal intensity and energy resolution. Such advancements would further deepen our understanding of vibrational energy redistribution dynamics and associated molecular processes by identifying the specific vibrational modes involved and their coupling in real‐space at the submolecular level.

Although mechanical anharmonicity carries more direct chemical significance, electrical anharmonicity and its spatial variation (Figure 3e) are also practically important, as they provide insight into strategies for enhancing the intensity of overtones and combination bands in experimental spectra (see Section S5).

## Conclusion

In summary, we investigated the anharmonicity of perylene vibrations in single PMI molecules adsorbed on Si(111)‐7×7 using STM‐based point‐contact TERS, which enabled the submolecular‐level measurement of overtones and combination bands. We recorded the lateral‐position dependence of both mechanical and electrical anharmonicities within the molecules with ∼3 Å resolution. Mechanical anharmonicity was exemplified for the **C+D** combination band, where anharmonic coupling between **C** and **D** modes was observed exclusively at the anhydride side, while the imide side remained uncoupled. This suggests a site‐specific vibrational energy exchange channel between **C** and **D** modes, confined to the anhydride side of the molecule. For all other observed overtones and combinations, the potential energy curves were found to be nearly harmonic, indicating minimal mechanical anharmonicity even under the adsorption on the surface and the point‐contact formation with the tip. These pronounced overtones and combination bands were attributed to electrical anharmonicity. Our findings demonstrate the potential of overtone and combination band spectroscopy via TERS as a powerful tool for probing vibrational energy landscapes and energy exchange processes at submolecular resolution. This approach could offer a means to directly visualize vibrational energy transfer routes in real space, such as those through hydrogen bonds^[^ ^67^ ^]^ and along protein backbones,^[^ ^68^ ^]^ providing critical structural insights that are not accessed through time‐resolved measurements alone.

## Conflict of Interests

The authors declare no conflict of interest.

## Supporting information

Supporting Information

## Data Availability

The data that support the findings of this study are available from the corresponding author upon reasonable request.
