# Supplementary material for: Submolecular‐Resolution Probing of Vibrational Anharmonicity Using Tip‐Enhanced Raman Spectroscopy
Source: Angew Chem Int Ed Engl. 2025 Sep 25;64(46):e202514215. doi: 10.1002/anie.202514215 (PMC12603967; doi:10.1002/anie.202514215)
Supplement: Supplementary file 1 — Supporting Information [file ANIE-64-e202514215-s001.pdf]

Supporting Information for "Submolecular-resolution probing of vibrational anharmonicity using tip-enhanced Raman spectroscopy"

Youngwook Park,*[a] Ikutaro Hamada,[b] Martin Wolf,[a] Akitoshi Shiotari*[a]

Contents

1. Methods 3

2. Influence of the tip on vibrational anharmonicity 4

3. Experimental factors affecting overtone and combination intensity in TERS: Plasmon and laser wavelength 5

4. Mechanical and electrical anharmonicity of vibrations 6

5. Discussion on the source of large electrical anharmonicity 7

6. Supplementary Tables 8

Table S1. Overtones and combination bands of modes **A**–**C** in TERS of PMI recorded at the imide site (Figure 2a in the main text) 8

Table S2. Overtones and combination bands of modes **A**–**D** in TERS of PMI recorded at the anhydride site (Figure 2b in the main text) 9

Table S3. Overtones and combination bands of modes **A**–**D** in TERS of DiMe-PDI (Figure S7a in SI) 10

7. Supplementary Figures 11

Figure S1. Visual guide to overtone and combination band assignments in PMI/Si(111) TERS spectrum 11

Figure S2. Raman spectra of PMI (a,b) and DiMe-PDI (c) powders 12

Figure S3. Heatmaps of vibrational anharmonicity of PMI 13

Figure S4. TERS spectra acquired using different Ag tips for distinct PMI molecules 14

Figure S5. Spectral comparison between the imide and anhydride sites of PMI/Si(111) before TERS intensity normalization 15

Figure S6. Constant-current STM image of DiMe-PDI/Si(111) 16

Figure S7. Spectral assignment of overtones and combination bands in DiMe-PDI/Si(111) TERS spectrum 17

Figure S8. Visual guide to overtone and combination band assignments in DiMe-PDI/Si(111) TERS spectrum 18

[a] Dr. Y. Park*, Prof. Dr. M. Wolf, Dr. A. Shiotari*
Department of Physical Chemistry, Fritz-Haber Institute of the Max-Planck Society, Berlin, Germany
E-mail: park@fhi-berlin.mpg.de
shiotari@fhi-berlin.mpg.de

[b] Prof. Dr. Ikutaro Hamada
Department of Precision Engineering, Graduate School of Engineering, The University of Osaka, Suita, Japan

Figure S9. Model schematics of 2D PES of two vibrational modes 19
Figure S10. TERS spectra of PMI when partially and completely picked up by the tip 20
Figure S11. TERS spectra of DiMe-PDI and PTCDA when partially picked up by the tip 21
Figure S12. Overtones and combination bands observed with two different tips: LSP energy dependence and resonance Raman effect 22
Figure S13. Tip-height dependence of vibrational anharmonicity in DiMe-PDI 23
Figure S14. A series of TERS spectra of DiMe-PDI/Si(111), highlighting the abnormal background in Figure S13 24

References 25

1. Methods

STM and TERS experiments The experiments were conducted in a UHV chamber equipped with a low-temperature STM (modified UNISOKU USM-1400) at a sample temperature of 78 K. The sample holder was equipped with three-dimensional coarse piezo motors and a piezo tube scanner, while the STM tip was fixed in position. A bias voltage (V_{bias}) was applied to the sample, with the tip kept grounded.

The tip positioning for the TERS measurements was performed as follows. First, the lateral position was adjusted to the center of the target molecule based on its STM image, after which the current feedback loop was opened at $V_{\text{bias}} = 0.5\,\text{V}$ and $j_{\text{STM}} = 50\,\text{pA}$, unless otherwise specified. Subsequently, the V_{bias} was tuned to $-0.3\,\text{V}$ unless stated otherwise, and the tip was first adjusted laterally and then vertically to reach a predefined sampling location. This procedure ensured that the tip height remained consistent during lateral-tip-position-dependent measurements. As a continuous-wave visible laser source for TERS, a 532-nm solid-state laser was used with a nominal incident power of 5.6 mW, unless otherwise indicated. A p-polarized laser beam entered the UHV chamber through a laser-line filter and a fused silica window. The laser was precisely focused onto the STM tip apex using an Ag-coated parabolic mirror (focal length 8 mm), mounted on a five-axis piezoelectric (x, y, z, θ, and ϕ) within the STM system. Acquisition time for each TERS spectrum was 3 s.

For both TERS and STML measurements, the scattered light from the junction was collected by the parabolic mirror and directed to a grating spectrometer (Andor Shamrock 303i; 303 mm focal length) located outside the UHV chamber via a beam splitter. All TERS spectra were recorded using a 600 lines/mm grating, with input and output slit widths of 200 and 10 μm, respectively, unless otherwise specified. The spectrometer was calibrated using Ne emission lines, and calibration accuracy was further validated using the bulk Si phonon peak ($520.2\,\text{cm}^{-1}$)[1]. A long-pass filter was used for TERS with a suitable cutoff wavelength corresponding to the incident laser wavelength. For spectral analysis, baseline correction of each TERS spectrum was performed using asymmetric least square smoothing, followed by fitting the data with multiple Gaussian peaks. All spectra presented in the main text are background-subtracted; the corresponding raw spectra for Figures 2 and 3, prior to background subtraction and normalization, are provided in Figure S5.

Sample and tip preparation Si(111) sample plates (Siegert Wafer GmbH; As doped) were degassed at 1023 K and then flash-annealed multiple times at 1473 K in the UHV chamber. The cleanliness of the surface and the formation of the 7×7-reconstruction were confirmed by STM imaging. The molecules were loaded into a Knudsen-cell evaporator, which was thoroughly degassed under UHV conditions. PMI (synthesized as described in the SI of ref.[2]) and DiMe-PDI (Luminescence Technology Corp.) were evaporated at 568 K and 558 K, respectively, onto the clean Si(111)-7×7 surface at room temperature.

To prepare the STM tips, chemically etched Ag wires were processed by focused ion beam milling using Ga ions, which sharpened the tips and provided highly reproducible plasmonic properties. To tune the plasmon resonance energy profiles of the Ag tips, a clean Ag(111) surface was used for adjusting the tip apex. The tip was gently poked into the surface, and voltage pulses (a few volts for 0.1 s) were applied to the Ag–Ag junction to modify the plasmonic properties.

DFT calculations The geometry of PMI and DiMe-PDI molecule adsorbed on the Si(111)- 7×7 corner hole was optimized using essentially the same method as described in our previous work[3]. In short, all the calculations were performed using the STATE code[4], which employs pseudopotentials to describe electron–ion interactions and plane-wave basis set to expand wavefunctions and charge density. The rev-vdW-DF2[5] was used for the exchange-correlation functional. PMI and DiMe-PDI molecules were put one-side of the Si(111) slab and the effective screening medium method[6,7] was used to eliminate spurious electrostatic interaction between the neighboring images.

Vibrational analyses, including the determination of atomic displacements, frequencies, Raman intensities, and construction of 2D PES, were performed at the B3LYP/6-31G(d) level using the Gaussian 16 suite of programs[8]. For these vibrational calculations, only the molecule, using the geometry optimized on the surface, was considered, with Si atoms excluded. This molecule-only approach, following initial optimization with the surface included, has been adopted in a prior study to simulate TERS spectra on Si(111)[9]. No imaginary vibrational modes were found in the calculations. To construct the 2D PES shown in Figure 5, we calculated the energy of the molecule as a function of displacements along the combined coordinates of two normal modes (i.e., modes **C1** and **D** for Figure 5a; modes **C2** and **D** for Figure 5b). For the calculated spectra in Figure 2c and others, Raman shift was scaled by a factor of 0.96, consistent with the commonly accepted scaling factor of 0.962 at the B3LYP/6-31G(d) level[10].

2. Influence of the tip on vibrational anharmonicity

To investigate the influence of the tip on vibrational anharmonicity, the Raman scattering from DiMe-PDI was examined as a function of gap distance d and tip position above the molecule. Tip approach–retraction cycles were conducted at four different positions within the molecule, labeled **1**–**4** in the molecular structure of DiMe-PDI in Figure S13a. STM current traces are shown in Figure S13b, which determine the contact regime indicated by the grey shading in Figures S13c–S13h.

Figures S13c–S13e show the Raman shifts of **D** and **2D** as the tip height is varied at different tip locations. The fundamental frequency of **D** was reversibly tuned by approximately $10\,\mathrm{cm}^{-1}$ during the tip approach and retraction cycle, reflecting a change in the curvature of the potential curve. This change was most pronounced at the onset of tip–molecule contact formation ($d = 0$), where the tip–molecule interaction increased dramatically [11]. Further downward movement of the tip beyond contact formation ($d \leq 0$) did not significantly affect the frequency. Throughout this change in fundamental frequency, the Raman shift of **2D** consistently tracked twice the frequency of **D**, indicating that the harmonic shape of the potential curve for mode **D** was not perturbed by the tip contact. This further confirms that the point-contact mode of TERS does not induce mechanical anharmonicity in the molecule, but simply amplifies the intensity of overtones and combination bands to enable reliable identification, as shown in Figure 1 in the main text.

Figures S13f–S13h present how peak intensities evolve with tip height at different molecular sites. Notably, two key features stand out in the $A_{\mathbf{2D}}/A_{\mathbf{D}}$ ratio in Figure S13h. First, an increasing trend in the $A_{\mathbf{2D}}/A_{\mathbf{D}}$ ratio was observed as the tip moved from the perylene body (position **2**) toward the methyl imide group (position **4**), similar to the trend observed in PMI. Second, the progression of the $A_{\mathbf{2D}}/A_{\mathbf{D}}$ ratio with tip height exhibited distinct patterns depending on the position: at position **2**, the ratio decreased as the tip height lowered, while at position **4**, it increased. Figure S13 enables the examination of the potential curve and electrical anharmonicity of the molecule under the influence of systematically controlled local environments at the atomic scale.

3. Experimental factors affecting overtone and combination intensity in TERS: Plasmon and laser wavelength

The intensity of overtones and combination bands in our TERS spectra could be affected by the nanostructure of the plasmonic tip, as the efficiency of tip-enhanced Raman scattering is influenced by the spectral profile of the localized surface plasmon (LSP) resonance of the junction. To evaluate this, we recorded TERS spectra of perylene-3,4,9,10-tetracarboxylic dianhydride (PTCDA) on the Si(111) surface using different Ag tips and different laser sources. The PTCDA/Si(111) sample was prepared in the same manner as described in ref. [3]. As demonstrated in Figures S12a–S12d, the use of two tips with distinct LSP resonance profiles resulted in markedly different relative intensities for the overtones in the same system. This observation underscores the potential to engineer the nanostructure of the STM tip apex in such a way to optimize preferential detection of high-energy transitions, enabling the observation of not only the first, but also higher order overtone and combination bands in single molecules (Figure S12d). The electromagnetic field gradient may also contribute to signal enhancement [12]; however, it alone cannot explain the intensity difference observed between the anhydride and imide sides of PMI/Si(111) (Figure S5), as the plasmonic gradient arising from the tip and surface structure is expected to be essentially identical on both sides.

We also observed that the relative overtone intensities vary significantly with the wavelength of the incident laser (Figures S12d and S12f). This behavior is likely influenced by the plasmonic profile of the junction. Additionally, the resonance Raman effect, which arises from the resonance between the incident laser and the electronic transitions of the molecules, may also contribute to this wavelength dependence. In our measurements, the electronic structure of the molecules is complicated due to their binding to both the surface and the tip (Figure S12e), making it challenging to precisely investigate the resonance effects. The resonance Raman effect in TERS has previously been observed for fundamental vibrational transitions of a single molecule on an insulating film over a metal surface [13]. A future challenge will be to explore whether electronic resonances in decoupled environments can also enhance overtones and combination bands, similar to conventional resonance Raman spectroscopy, thereby providing deeper insights into the underlying TERS mechanisms.

4. Mechanical and electrical anharmonicity of vibrations

The polarization p of the molecule with polarizability α under the influence of electric field E of light could be expressed for a specific normal mode n (with its coordinate Q_n)[14],

$$\begin{aligned} p &= \alpha E \\ &= \alpha E_0 \cos(\omega t) \\ &= \left[\alpha_0 + \sum_n \left(\frac{\partial \alpha}{\partial Q_n}\right)\bigg|_0 Q_n\right] E_0 \cos(\omega t) \\ &= \left[\alpha_0 + \sum_n \left(\frac{\partial \alpha}{\partial Q_n}\right)\bigg|_0 Q_{n_0} \cos(\omega_n t + \delta_n)\right] E_0 \cos(\omega t), \end{aligned} \tag{1}$$

where ω_n and ω correspond to the frequencies of the mode n and of incident light, respectively, and δ_n represents the phase of n. The Stokes Raman transition appears at $\omega - \omega_n$. Two anharmonic contributions are extracted from the formula; one is mechanical anharmonicity, and the other is commonly called electrical anharmonicity. The mechanical anharmonicity is the deviation of the normal mode from a harmonic oscillator.

$$Q_n = Q_{n_0} \cos(\omega_n t) + Q_{n_0}^{(2)} \cos(2\omega_n t) + \cdots . \tag{2}$$

The vibrational energy ν incorporating mechanical anharmonicity is expressed as:

$$\nu = \sum_n \nu_n \left(v_n + \frac{1}{2}\right) + \sum_{m \geq n} \chi_{nm} \left(v_n + \frac{1}{2}\right)\left(v_m + \frac{1}{2}\right). \tag{3}$$

Here, ν_n represents the frequency of mode n, v_n denotes the vibrational quantum number, and χ_{nm} is the anharmonicity constant ($m = n$ for overtones, $m > n$ for combinations). Notably, the anharmonicity constants are negative, leading to the downshift. This downshift is typically minor, only a few percent relative to the expected transition energy. Mechanical superharmonicity, a concept that contrasts with mechanical anharmonicity, can lead to larger energy separations (positive anharmonicity constants) for overtones compared to the fundamental transitions[15,16]. However, this effect is generally associated with highly anharmonic potentials dominated by significant quartic terms, often observed in systems with strong hydrogen bonds[17]. Such behavior is unlikely to be present in the intramolecular vibrational modes **A**–**D** of PMI and DiMe-PDI.

The electrical anharmonicity comes from the second or higher derivatives of the polarizability along the vibrational coordinates;

$$\alpha = \alpha_0 + \sum_n \left(\frac{\partial \alpha}{\partial Q_n}\right)\bigg|_0 Q_n + \sum_{m \geq n} \left(\frac{\partial^2 \alpha}{\partial Q_n \partial Q_m}\right)\bigg|_0 Q_n Q_m + \cdots . \tag{4}$$

An overtone or a combination band could be detected even for a perfect harmonic oscillator or uncoupled vibrations when the nonlinear terms in the expansion are non-zero.

5. Discussion on the source of large electrical anharmonicity

Here, we discuss the source of the extraordinarily high relative intensity of overtones and combination bands observed in this work, exceeding 0.2 at the PMI imide site (Figure 3e). This remarkable intensity, observed for both PMI and DiMe-PDI on Si(111), particularly at the molecular edges, is of electrical origin, as most vibrations were found to be mechanically harmonic (Figure 4a). Unraveling the underlying mechanism behind the enhancement of overtones and combinations at the submolecular scale, within a well-characterized molecular configuration and molecule–surface bonding environments, could improve our understanding of the TERS process by linking the scattering site to the observed intensity. Empirically, we hypothesize that the binding configuration of the molecules to the surface plays a significant role in the enhancement of polarization, which enables the detection of intrinsically weak transitions. The O–Si bonds, oriented nearly perpendicular to the surface (Figure 1a)[3], may act as antennas for polarization in the surface-normal direction.

The observed site dependence of the overtone intensities suggests that the O–Si bonds are key to the enhancement mechanism.

1. $A_{\mathbf{2D}}/A_{\mathbf{D}}$ is always larger above the imide or anhydride group (Figures 3e and S13h), where the molecule forms O–Si bonds with the surface, compared to the center of PMI and DiMe-PDI molecules. Additionally, although less prominently than the trend along the molecular long axis, the overtone intensity increases at the carbonyl O atom compared to the central N or O atom, as shown in Figure S3b for PMI.

2. For PMI, $A_{\mathbf{2D}}/A_{\mathbf{D}}$ values are higher on the imide side than on the anhydride side (Figure 3e), which is consistent with the stronger binding affinity of the imide group to Si adatoms on the surface[3].

3. The position-dependent progression of $A_{\mathbf{2D}}/A_{\mathbf{D}}$ observed for DiMe-PDI (Figure S13h) can also be explained by changes in surface binding. As the tip approaches after initial contact formation ($d \leq 0$), the repulsive tip–molecule interaction pushes the molecule downward. When the tip presses directly on the imide group (position **4** in Figure S13), the O–Si bonds are strengthened, leading to an increasing trend in the overtone intensity. In contrast, when the tip presses away from the imide group (position **2** in Figure S13), the O–Si bonds may weaken due to the bending of the molecular frame, leading to a decreasing trend in $A_{\mathbf{2D}}/A_{\mathbf{D}}$.

4. When the molecule is partially or fully detached from the surface by the tip—resulting in the loss of some or all O–Si bonds—no site dependence of $A_{\mathbf{2D}}/A_{\mathbf{D}}$ was observed (Figure S10).

As depicted in Figures 2d and S7c, the normal modes **A**–**D** do not involve the motion of the carbonyl O atoms. However, it is important to note that an atom does not necessarily need to physically move during a vibration for Raman signals to be detected[18]; instead, changes in electrical polarizability are required. We propose that the observed signals in the overtones and combinations arise from higher-order polarization occurring during the intramolecular vibrations, which are particularly enhanced near the carbonyl O atoms that form nearly vertical O–Si bonds with the surface. Elucidating the detailed underlying mechanism remains a task for future investigation.

6. Supplementary Tables

Table S1. Overtones and combination bands of modes **A–C** in TERS of PMI recorded at the imide site (Figure 2a in the main text). Peak positions and intensities were determined by Gaussian peak fittings. Errors are the standard deviations of the fittings.

Raman shift (cm^{-1})	Symbol	Δ_{i+j} (cm^{-1})	$A_{over,comb}/A_{fund}$ [a]
1236.9 ± 1.2	**A**		
1270.7 ± 1.1	**B**		
1359.9 ± 1.3	**C**		
1602.7 ± 0.2	**D**		
2475.0 ± 1.8	**2A**	1.3 ± 2.5	0.25 ± 0.05
2515.3 ± 2.5	**A+B**	7.8 ± 3.0	0.16 ± 0.02
2604.4 ± 4.1	**A+C**	7.6 ± 4.4	0.24 ± 0.06
2841.0 ± 0.9	**A+D**	1.4 ± 1.5	0.17 ± 0.01
2876.2 ± 1.7	**B+D**	2.8 ± 2.1	0.06 ± 0.01
2961.2 ± 1.6	**C+D**	−1.4± 2.1	0.13 ± 0.01
3201.1 ± 0.9	**2D**	−4.3± 0.9	0.22 ± 0.01

[a] For an overtone, A_{over}/A_{fund}, where A_{over} and A_{fund} are the peak areas of the overtone and fundamental peaks, respectively. For a combination band, $A_{comb}/(A_{fund1} + A_{fund2})$, where A_{comb} is the combination peak area and A_{fund1} and A_{fund2} are the areas of two contributing fundamentals. Same applies to Tables S2 and S3.

Table S2. Overtones and combination bands of modes **A–D** in TERS of PMI recorded at the anhydride site (Figure 2b in the main text).

Raman shift (cm^{-1})	Symbol	Δ_{i+j} (cm^{-1})	$A_{\text{over,comb}}/A_{\text{fund}}$
1235.0 ± 0.6	**A**		
1279.1 ± 1.8	**B**		
1380.4 ± 0.4	**C**		
1604.8 ± 0.1	**D**		
2472.8 ± 2.4	**2A**	2.8 ± 2.6	0.07 ± 0.01
2525.0 ± 3.6	**A+B**	10.9 ± 4.1	0.05 ± 0.01
2612.7 ± 2.3	**A+C**	−2.7± 2.4	0.066 ± 0.006
2841.3 ± 0.7	**A+D**	1.5 ± 0.9	0.085 ± 0.004
2883.2 ± 2.4	**B+D**	−0.8± 3.0	0.019 ± 0.004
2974.5 ± 1.7	**C+D**	−10.7± 1.8	0.031 ± 0.003
3208.9 ± 1.2	**2D**	−0.7± 1.2	0.087 ± 0.005

Table S3. Overtones and combination bands of modes **A–D** in TERS of DiMe-PDI (Figure S7a in SI).

Raman shift (cm^{-1})	Symbol	Δ_{i+j} (cm^{-1})	$A_{\mathrm{over,comb}}/A_{\mathrm{fund}}$
1236.8 ± 0.4	**A**		
1369.9 ± 0.8	**C**		
1601.8 ± 0.2	**D**		
2474.6 ± 2.1	**2A**	1.0 ± 2.2	0.17 ± 0.03
2605.5 ± 1.2	**A+C**	−1.2 ± 1.5	0.12 ± 0.02
2837.7 ± 1.2	**A+D**	−0.9 ± 1.3	0.20 ± 0.02
2970.4 ± 1.7	**C+D**	−1.3 ± 1.9	0.13 ± 0.02
3202.8 ± 2.3	**2D**	−0.8 ± 2.3	0.16 ± 0.02

7. Supplementary Figures

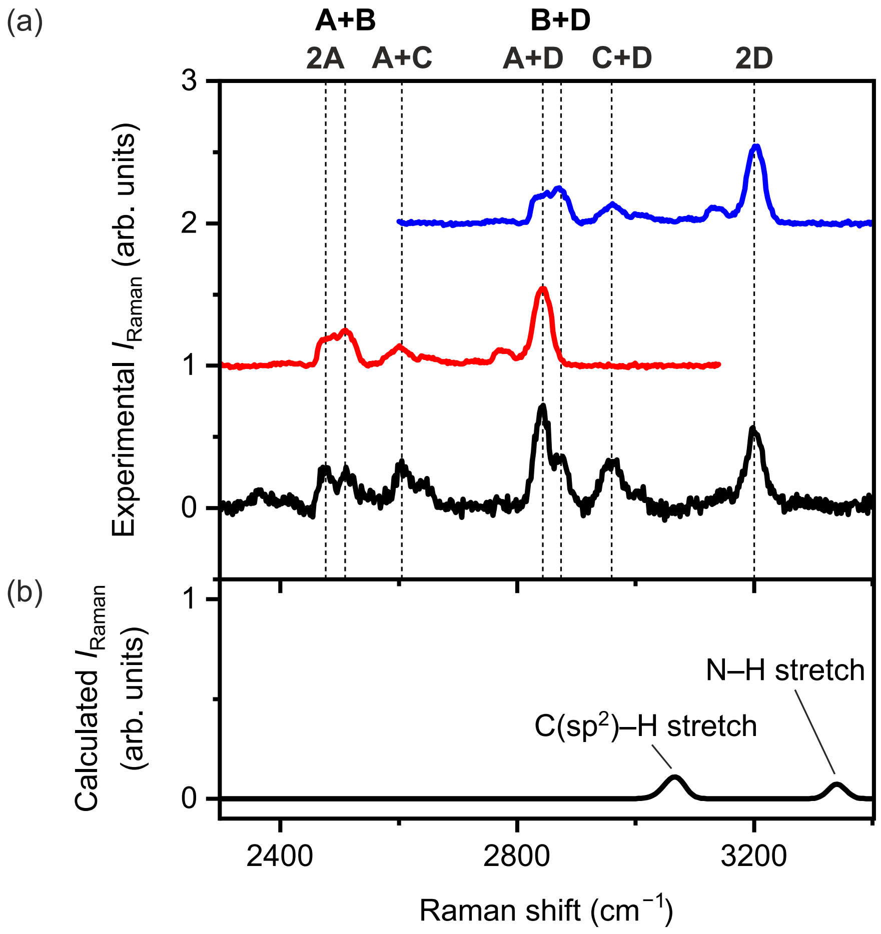

Figure S1. Visual guide to overtone and combination band assignments in PMI/Si(111) TERS spectrum. (a) Experimental TERS spectrum of overtones and combination bands of PMI/Si(111) recorded at the imide N atom (black), shown together with the fundamental transitions horizontally shifted by the frequency of **A** (red) and **D** (blue). The spectrum is identical to that shown in Figure 2a of the main text. (b) Calculated Raman spectrum of PMI, identical to that shown in Figure 2c of the main text. Comparison of the overtone and combination band positions with the shifted fundamental peaks, as well as with the calculated Raman shift of the C-H stretch, confirms the assignments.

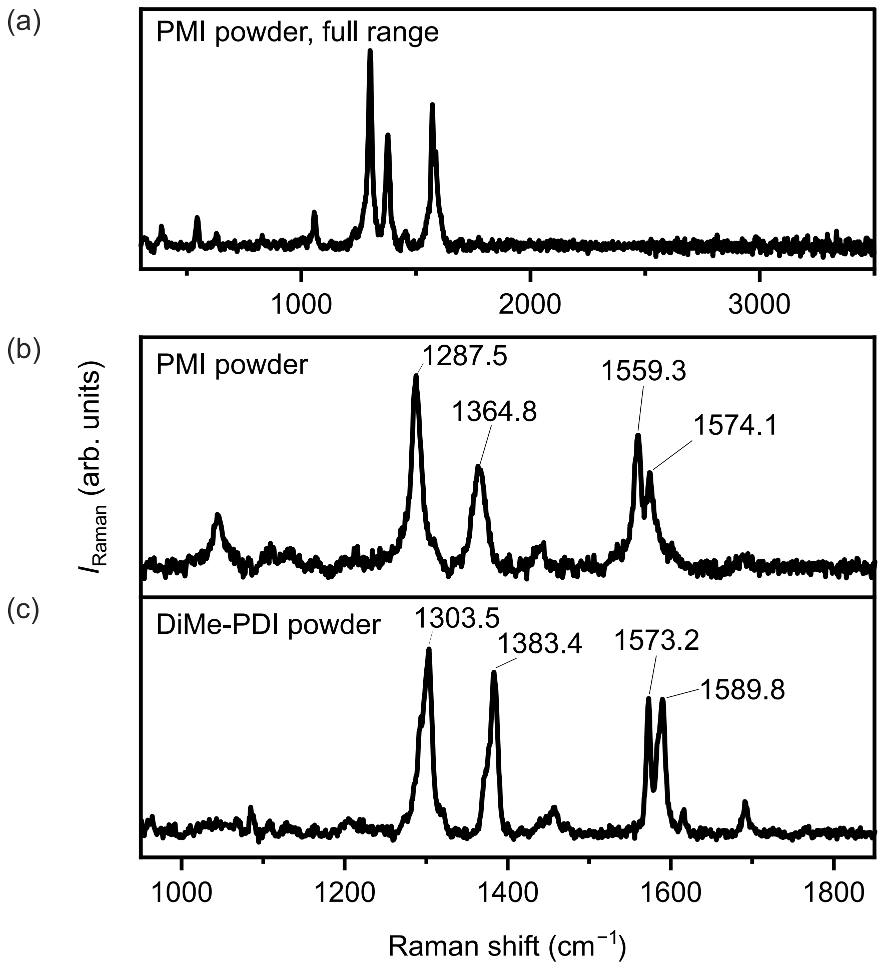

Figure S2. Raman spectra of PMI (a,b) and DiMe-PDI (c) powders. The spectra were acquired at room temperature using a Raman microscope (Horiba XploRA) with acquisition times of 60 and 50 seconds, respectively. A 785 nm laser was used under a parallel polarization detection scheme. Baselines were corrected by asymmetric least square smoothing. The full-range Raman spectrum (not shown) of DiMe-PDI, like that of PMI (a), also revealed no peaks in the high wavenumber region above 2000 cm^{-1}.

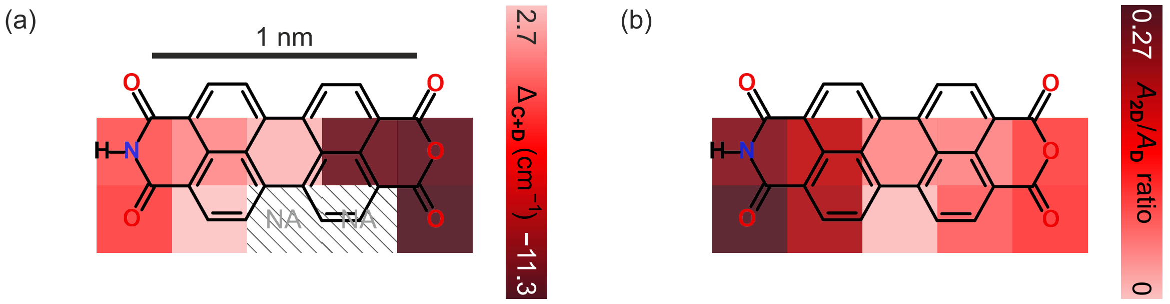

(c) Δ_{C+D} (cm^{-1})

−1.4 (±2.1)	0.6 (±2.6)	2.2 (±5.6)	−10.1 (±5.3)	−10.7 (±1.8)
−2.2 (±2.3)	2.7 (±7.3)	NA	NA	−11.3 (±1.6)

(d) A_{2D}/A_D ratio

0.23 (±0.01)	0.18 (±0.01)	0.04 (±0.02)	0.05 (±0.02)	0.09 (±0.01)
0.27 (±0.05)	0.20 (±0.07)	0.006 (±0.005)	0.07 (±0.04)	0.10 (±0.04)

Figure S3. Heatmaps of vibrational anharmonicity of PMI. (a) Heatmap of $\Delta_{\mathrm{C+D}}$. "NA" denotes the tip positions where the value was not available due to weak intensity of the combination band. (b) Heatmap of the $A_{\mathrm{2D}}/A_{\mathrm{D}}$ ratio. (c, d) Values and errors for (a) and (b), respectively.

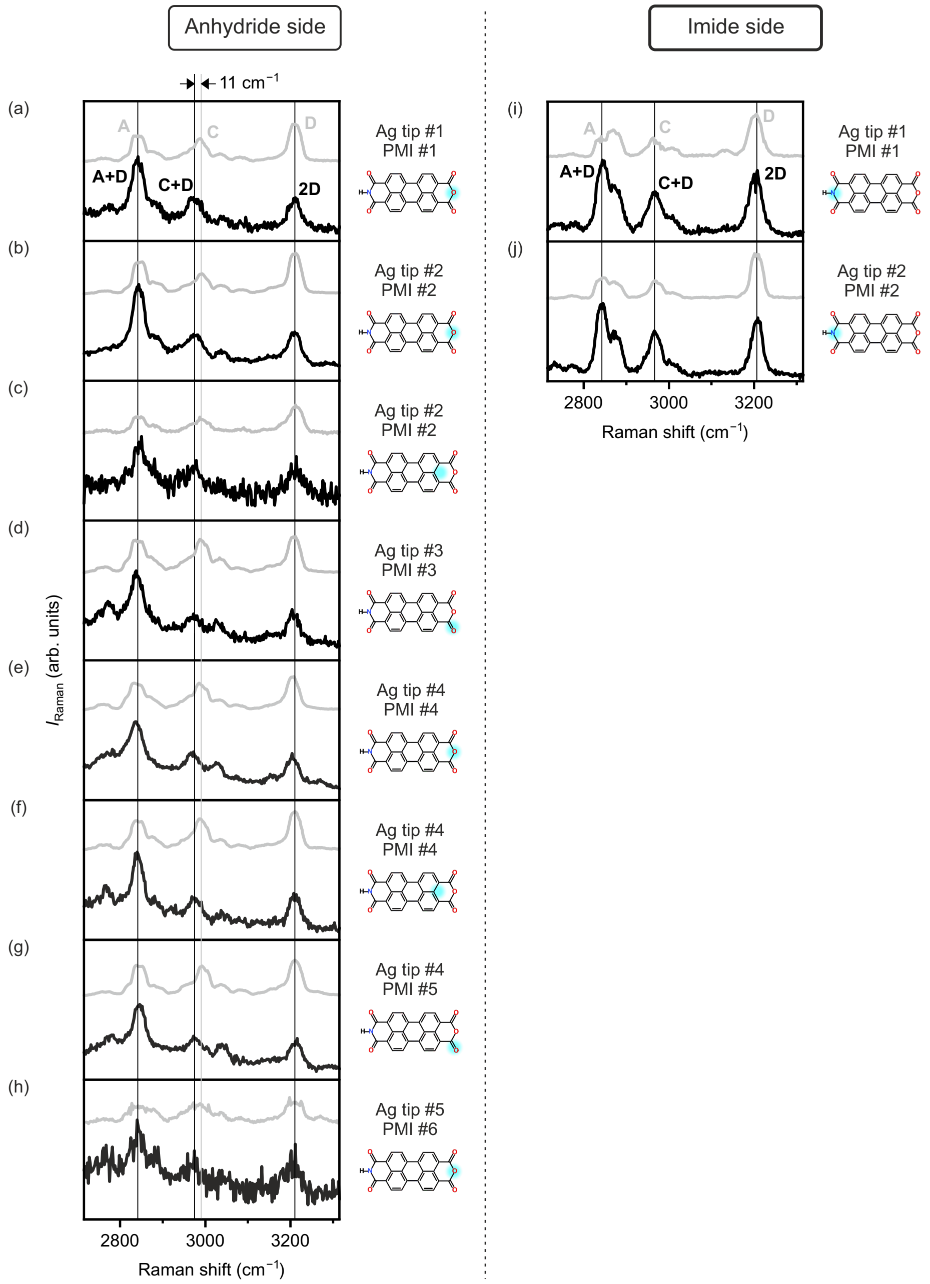

Figure S4. TERS spectra acquired using different Ag tips for distinct PMI molecules. (a–h) Anhydride side. (i,j) Imide side. Black lines indicate the overtones and combination bands, while gray lines represent the fundamental peaks laterally shifted by the energy of mode **D**. The intensity of fundamentals are scaled by 0.1 in (a–h) and 0.25 in (i,j). The spectra were obtained using varying Ag tips, PMI molecules, and tip positions within the molecules, as indicated on the right side of each plot. All anhydride-side spectra (a–h) consistently showed a $-11\,\mathrm{cm}^{-1}$ of **C+D** peak, confirming the reproducibility. Note that the Ag tips #1–#5 were different from the tip used for Figure 3 in the main text.

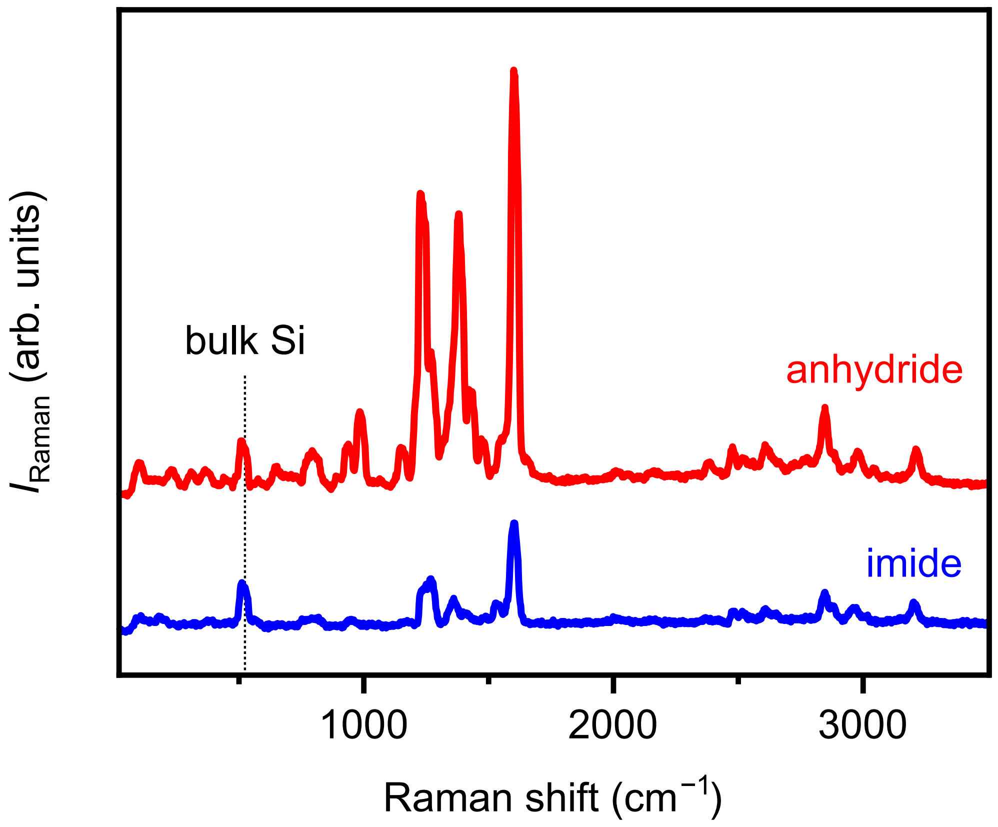

Figure S5. Spectral comparison between the imide and anhydride sites of PMI/Si(111) before baseline subtraction and intensity normalization. Note the same intensity of the bulk Si phonon peak in both spectra at 520.2 cm^{-1}. These spectra are baseline-subtracted and normalized to the intensity of fundamental transition **D** to result in the spectra in Figures 2 and 3 in the main text.

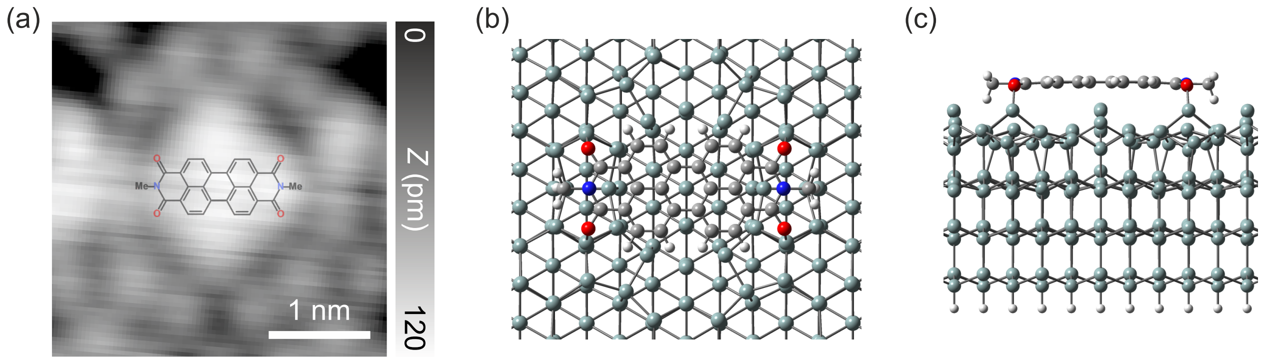

Figure S6. (a) Constant-current STM image of DiMe-PDI/Si(111). $V_{\rm bias} = 0.8\,\rm V$, $j_{\rm STM} = 50\,\rm pA$. The molecular structure of DiMe-PDI is overlaid on the image. (b, c) Top- and side-view of a calculated structure of DiMe-PDI/Si(111). The white, grey, blue, red and dark cyan spheres represent H, C, N, O and Si atoms, respectively.

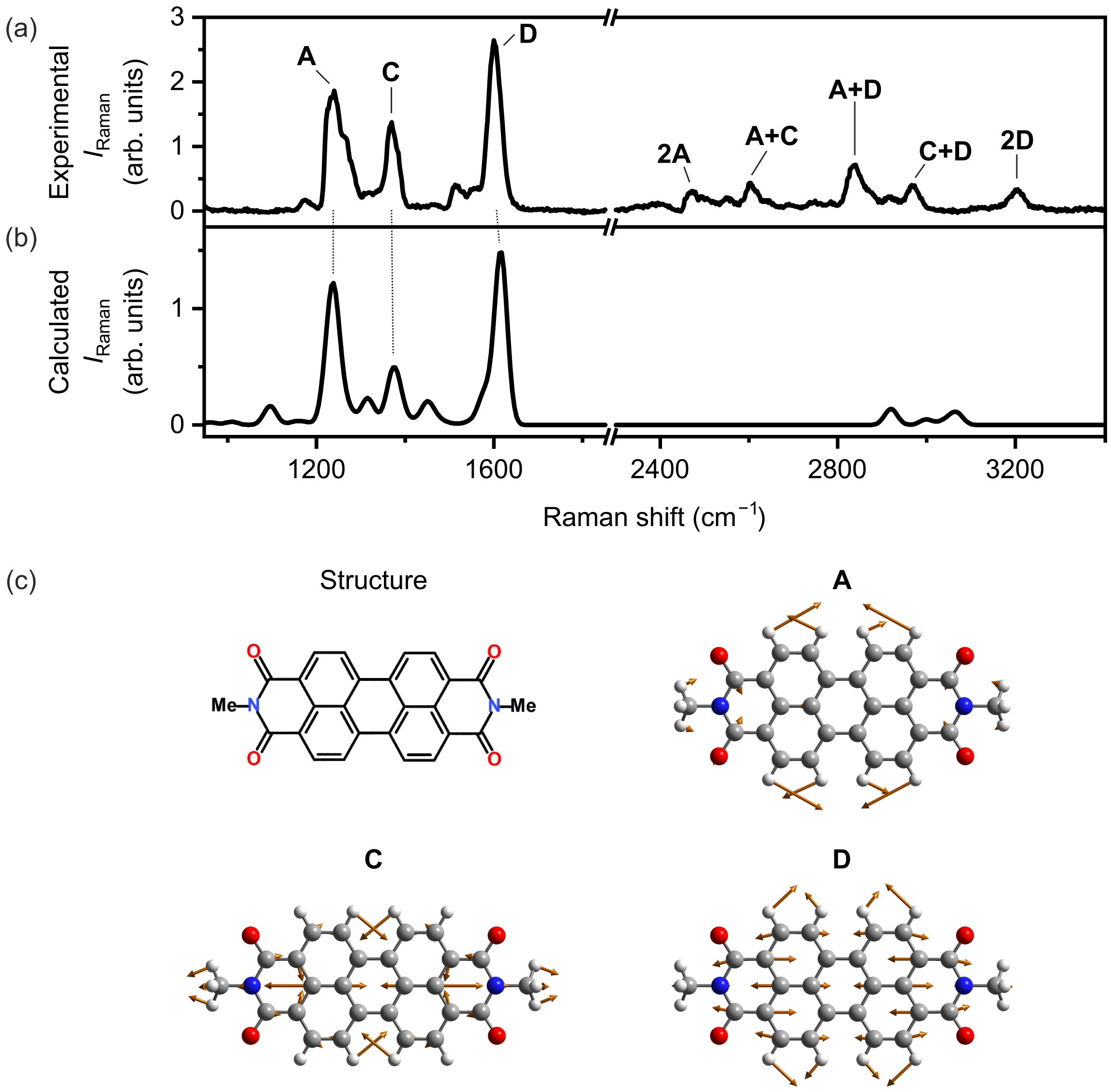

Figure S7. Spectral assignment of overtones and combination bands in DiMe-PDI/Si(111) TERS spectrum. (a) Experimental TERS spectrum of DiMe-PDI/Si(111) acquired at the N atom of the imide group. $d = -0.8$ Å. The splitting between **A** and **B** is less prominent compared to the spectrum of PMI/Si(111). (b) Calculated Raman spectrum of DiMe-PDI. (c) Molecular structure and calculated atomic displacements for the normal modes **A**, **C**, and **D** of DiMe-PDI.

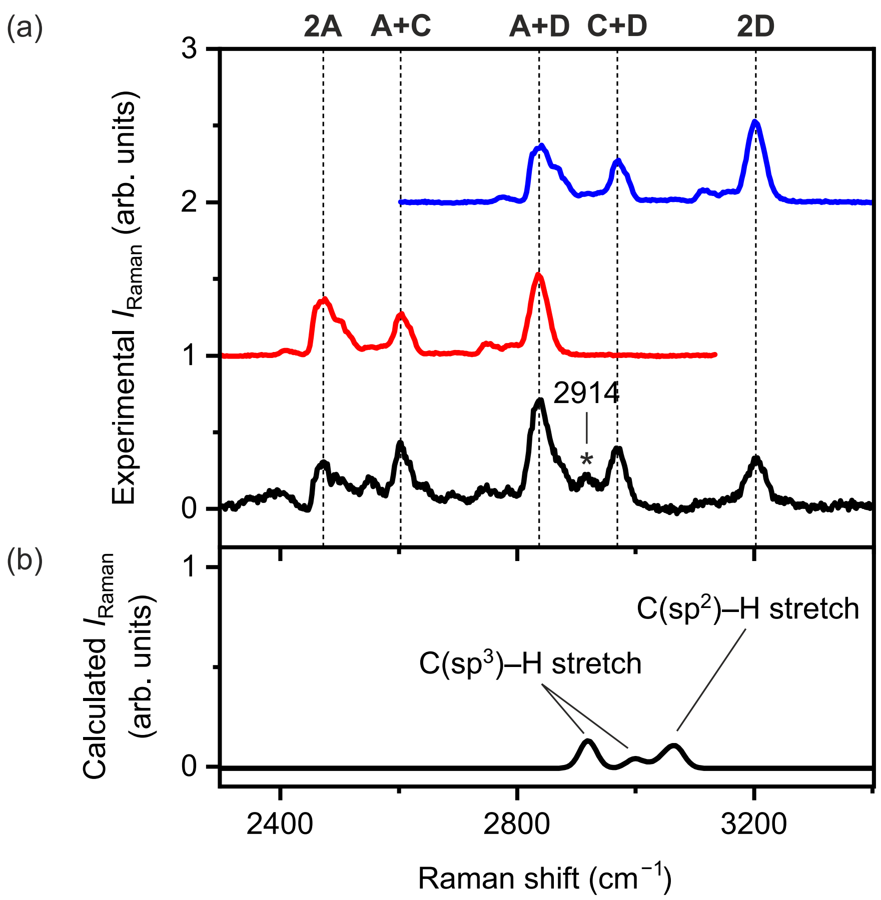

Figure S8. Visual guide to overtone and combination band assignments in DiMe-PDI/Si(111) TERS spectrum. (a) Experimental TERS spectrum of overtones and combination bands of DiMe-PDI/Si(111) recorded at the imide N atom (black), shown together with the fundamental transitions horizontally shifted by the frequency of **A** (red) and **D** (blue). The spectrum is identical to that shown in Figure S7a. (b) Calculated Raman spectrum of DiMe-PDI, identical to that shown in Figure S7b. Comparison of the overtone and combination band positions with the shifted fundamental peaks, as well as with the calculated Raman shift of the C-H stretch, confirms the assignments. The peak marked with an asterisk in (a) (at 2914 cm^{-1}) is likely attributed to the C-H stretching of the methyl group.

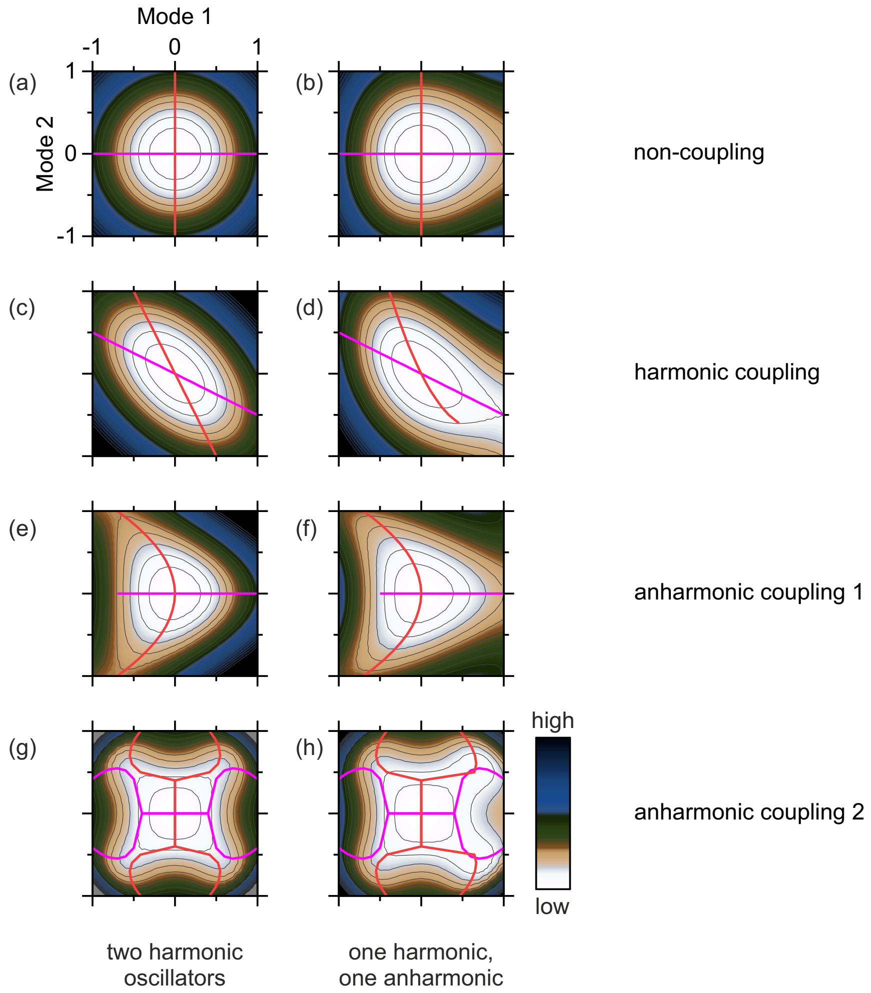

Figure S9. Model schematics of 2D PES of two vibrational modes. Left column shows PES for both modes being harmonic oscillators. In the right column, Mode 1 is an anharmonic oscillator while Mode 2 remains harmonic. (a, b) Non-coupling. (c, d) Harmonic coupling. (e, f) Anharmonic coupling type 1. (g, h) Anharmonic coupling type 2. Type 1 and Type 2 correspond to the cases where Mode 2 is coupled asymmetrically and symmetrically, respectively, with the displacement in Mode 1. Formula to depict each 2D PES are as follows: (a) x^2+y^2, (b) $x^2-0.5x^3+y^2$, (c) x^2+y^2+xy, (d) $x^2-0.5x^3+y^2+xy$, (e) $(x+0.7y^2)^2+y^2-(0.7y^2)^2$, (f) $(x+0.7y^2)^2-0.5(x+0.7y^2)^3+y^2-(0.7y^2)^2+0.5(0.7y^2)^3$, (g) $x^2+y^2+4y^2(-0.8x^2+x^4)+4x^2(-0.8y^2+y^4)$ and (h) $x^2-0.5x^3+y^2+4y^2(-0.8x^2+x^4)+4x^2(-0.8y^2+y^4)$, where x and y corresponds to the Modes 1 and 2 coordinates, respectively. Red and magenta curves show the energy minimum coordinates, as in Figure 5 in the main text.

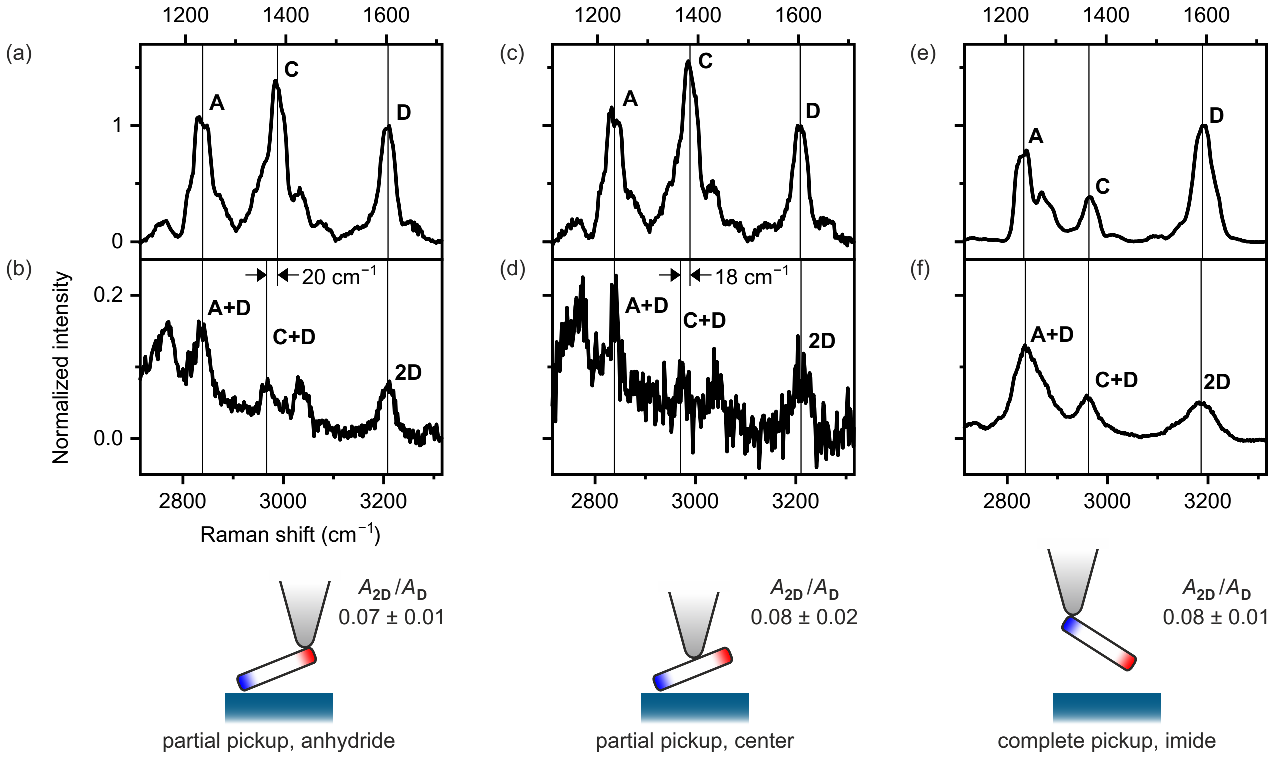

Figure S10. TERS spectra of PMI when partially and completely picked up by the tip. (a, b) Fundamental (a) and overtone/combination (b) transitions of PMI with the tip partially picking up the molecule at its anhydride group. Z_{rel} (relative tip height from the setpoint) = -4.3 Å ($d = 1.2$ Å). (c, d) Partially picked-up PMI with the tip at the center of the molecule. $Z_{\mathrm{rel}} = -4.3$ Å ($d = 1.2$ Å). The PMI molecule was partially picked up by the tip when the tip was positioned above the anhydride side or the center of the molecule, with d adjusted to 1–2 Å [3]. This partial pickup state was experimentally confirmed by reversible switching in STM current and changes in TERS intensities. For partial pickup PMI, the imide group still forms the O–Si bonds with the surface. (e, f) Fully picked-up PMI at imide site. $Z_{\mathrm{rel}} = -1.5$ Å ($d = 4.0$ Å). The PMI was fully picked up by the tip, though rarely, when the tip was positioned above the N atom in the imide group and retracted from the tip–molecule contact. The full pickup was identified by a dramatic change in STM current and the absence of the molecule in consecutive STM images. All spectra are normalized to the intensity of the mode **D**. At the bottom of each plots, $A_{\mathbf{2D}}/A_{\mathbf{D}}$ ratio is provided together with a schematic of the junction during TERS acquisition. All spectra were acquired using the same Ag tip.

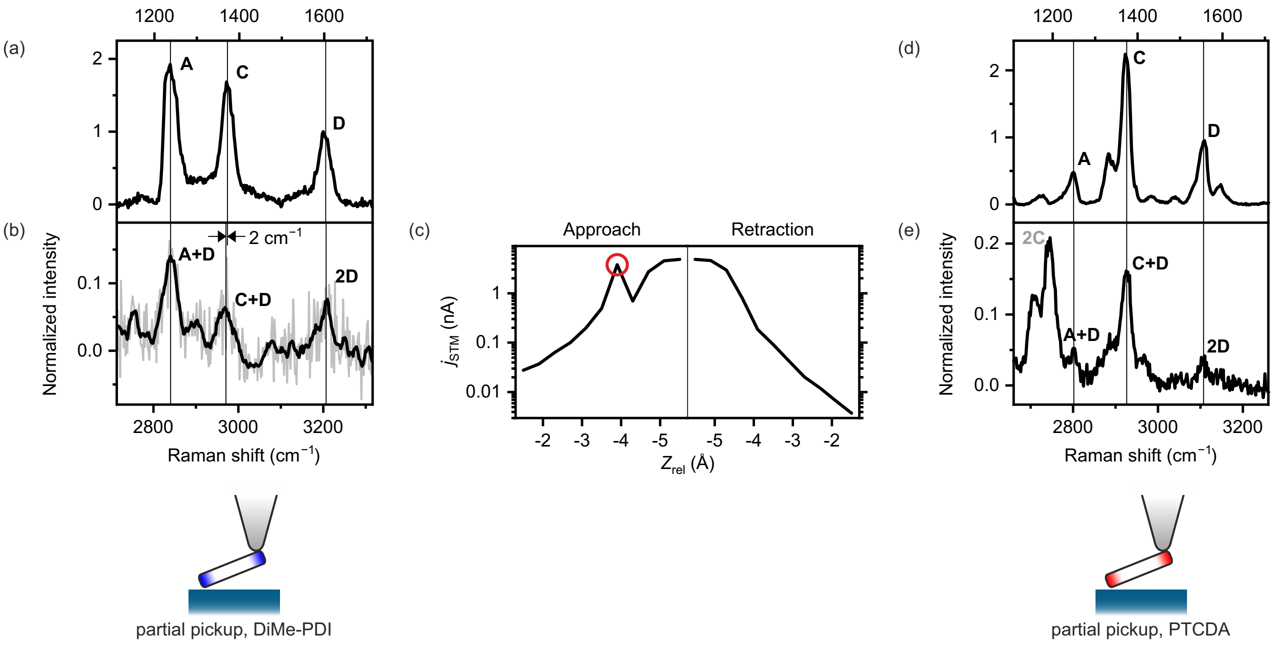

Figure S11. TERS spectra of DiMe-PDI and PTCDA when partially picked up by the tip. (a, b) Fundamental (a) and overtone/combination (b) transitions of DiMe-PDI with the tip partially picking up the molecule at its methyl imide group. The spectrum is normalized to the intensity of the mode **D**. (c) STM current of DiMe-PDI/Si(111) as a function of Z_{rel}. The spike (marked with red circle) during the tip approach indicates the partial pickup of DiMe-PDI, similar to what was observed with PTCDA and PMI [3]. The spectra shown in (a) and (b) were acquired during this partial pickup event, $Z_{\mathrm{rel}} = -3.9$ Å ($d = 0.8$ Å). The partial pickup did not occur every time even under the identical tip height; when the same tip was retracted from the surface, as shown in the right part of (c), the molecule remained adsorbed on the surface. Also, the partial pickup did not happen during the tip approach–retraction cycles for Figure S13, implying a low chance of the pickup event. Confirming the conditions for the partial pickup remains a subject for future investigation. (d, e) Fundamental (d) and overtone/combination (e) transitions of PTCDA partially lifted by the tip at its anhydride group. The PTCDA/Si(111) sample was prepared in the same manner as described in ref. [3]. Feedback opened at $V_{\mathrm{bias}} = 0.3$ V and $j_{\mathrm{STM}} = 50$ pA, $Z_{\mathrm{rel}} = -3.9$ Å ($d = 1.5$ Å).

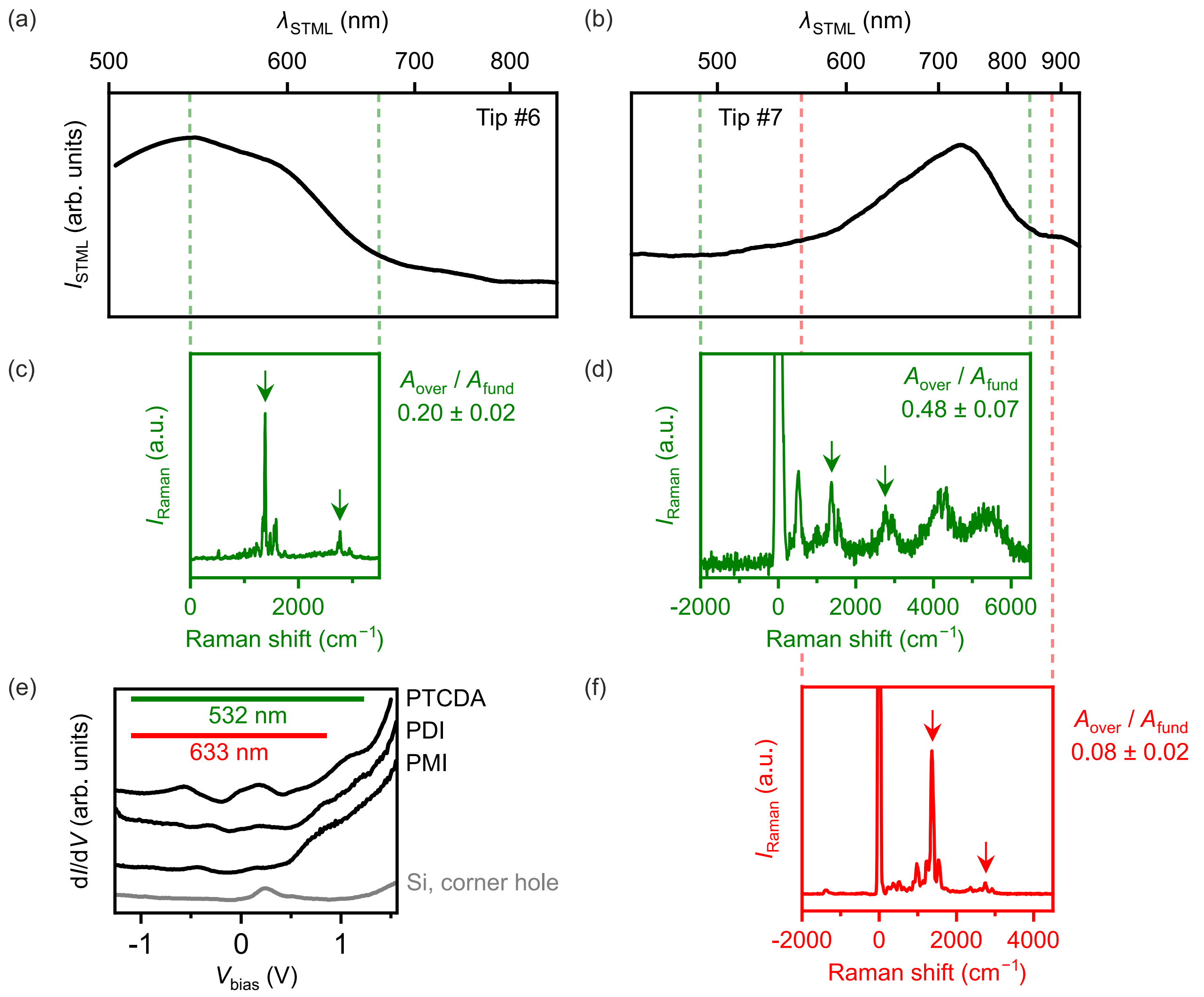

Figure S12. Overtones and combination bands observed with two different tips: LSP energy dependence and resonance Raman effect. (a, b) STM-induced luminescence (STML) measured on bare Si(111) surfaces with two different tips, namely Tips #6 and #7, representing distinct LSP resonance. V_{bias} = 3 V for both, j_{STM} = 10 and 1 nA for (a) and (b), respectively, with feedback loops closed. Acquisition time = 30 s. STML spectra were recorded without laser irradiation. (c, d) TERS spectra acquired with the Tips #6 and #7, respectively, for single PTCDA molecules adsorbed on Si(111). Feedback opened at V_{bias} = 0.3 V and j_{STM} = 50 pA, $Z_{\mathrm{rel}} = -3.9$ Å (d = 1.5 Å) (c) and -3.3 Å (d = 2.1 Å) (d). $A_{\mathrm{over}}/A_{\mathrm{fund}}$ ratios for the strongest fundamental (~1380 cm^{-1}) and its first overtone (~2760 cm^{-1}) transitions, marked with arrows, are shown together with the spectra. Note that in (d) the second overtone appears at ~4140 cm^{-1}. TERS spectra in (c) and (d) are displayed with the wavelength matched with the STML in (a) and (b), respectively, so that the wavelengths of the observed Raman scattering can be readily indicated in the LSP resonance energy profile. (e) Scanning tunneling spectra for PTCDA, PDI, and PMI molecules on Si(111), together with the spectrum recorded on a bare corner hole of the surface. The spectra were recorded by lock-in detection (21 mV$_{\mathrm{rms}}$ at 987 Hz) with the current feedback open (set-point current = 50 pA, V_{bias} = 0.5 V). The horizontal bars on top of the spectra indicate the photon energy scales of 532 nm (upper) and 633 nm (lower) as references. (f) TERS spectrum acquired with the Tip #7 for the PTCDA/Si(111) measured with incident laser (source: HeNe) wavelength of 633 nm (power = 9.7 mW). Feedback opened at V_{bias} = 0.3 V and j_{STM} = 50 pA, $Z_{\mathrm{rel}} = -4.2$ Å (d = 1.2 Å). Comparison of (f) with (d) provides the incident-wavelength dependence of $A_{\mathrm{over}}/A_{\mathrm{fund}}$. At the tip heights where (c), (d) and (f) were recorded, the PTCDA molecule undergoes reversible tip–molecule contact formation and dissociation, accompanied by the breaking of O–Si bonds. Raman signals emerge only when the molecule is in contact with the tip (partial pickup)[3]. The STML spectra in (a) and (b) and the TERS spectrum in (d) were recorded using a 150 lines/mm grating. The TERS spectra in (c), (d), and (f) are raw spectra, without baseline subtraction or normalization.

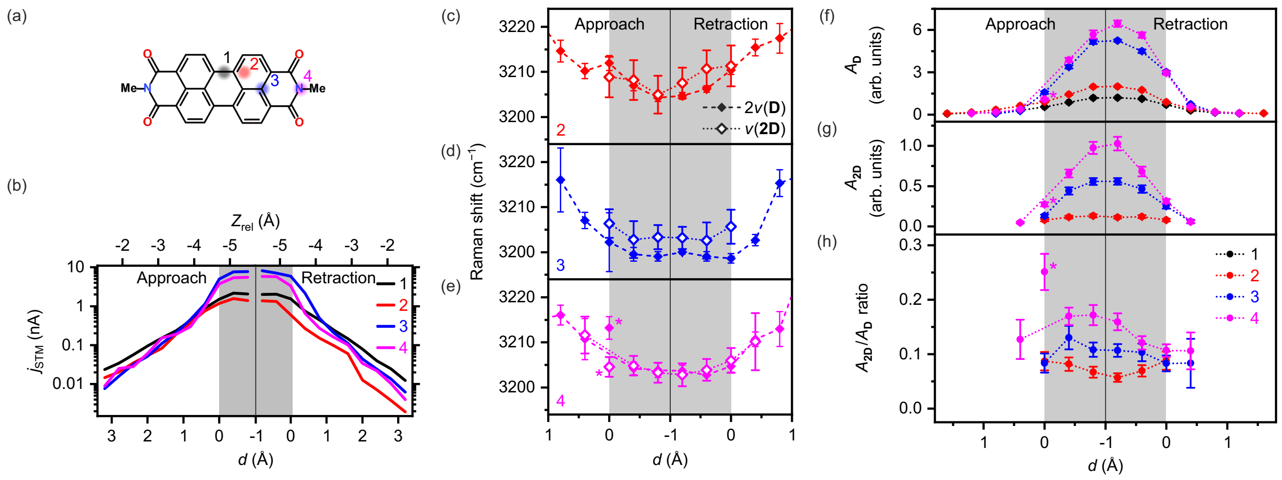

Figure S13. Tip-height dependence of vibrational anharmonicity in DiMe-PDI. (a) Molecular structure of DiMe-PDI, with the tip locations where the TERS spectra were acquired marked with color-matched circles. (b) STM current with the tip approaching and retracting at the four locations on DiMe-PDI marked in (a). Contact regime determined by the STM current saturation ($d \leq 0$) is shaded gray in (b)–(h). (c–e) Peak position of **D** and its first overtone **2D** at different tip locations 2 (c), 3 (d), and 4 (e), as a function of d. The peak positions of **D** fundamental are plotted doubled to compare with those of **2D**. (f–h) Integrated area of **D** (f), **2D** (g), and the ratio between two (h) as a function of d. At location 1, **2D** was not observed in the spectra. Peak position, integrated area, and their errors originate from Gaussian peak fittings. Note that the spectrum recorded at $d = 0$ Å during approach at tip location 4 (marked with asterisks in (e) and (f)–(h)) exhibited an abnormal background (see Figure S14) and was therefore excluded from the trend.

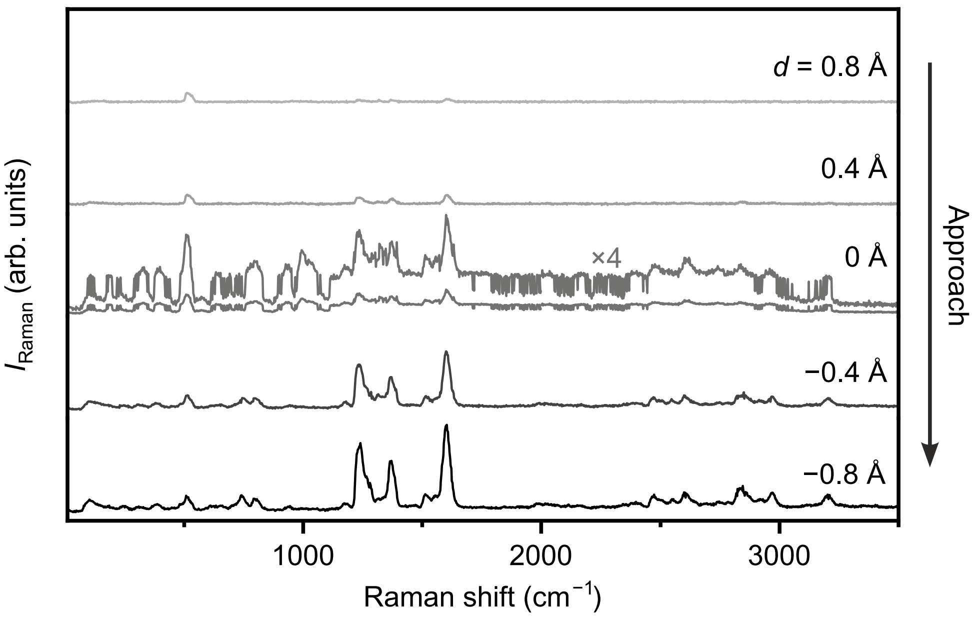

Figure S14. A series of TERS spectra of DiMe-PDI/Si(111), highlighting the abnormal background observed at $d = 0$ Å during tip approach at location 4 in Figure S13. The spectra are vertically offset for clarity, with the $d = 0$ Å spectrum shown alongside its $\times 4$ magnification.

References

[1] J. H. Parker Jr., D. W. Feldman, M. Ashkin, *Phys. Rev.* **1967**, *155*, 712.
[2] R. van der Weegen, P. A. Korevaar, P. Voudouris, I. K. Voets, T. F. A. de Greef, J. A. J. M. Vekemans, E. W. Meijer, *Chem. Commun.* **2013**, *49*, 5532.
[3] Y. Park, I. Hamada, A. Hammud, T. Kumagai, M. Wolf, A. Shiotari, *Nat. Commun.* **2024**, *15*, 6709.
[4] Simulation Tool for Atom Technology (STATE), https://state-doc.readthedocs.io/, accessed: October 25, 2023.
[5] I. Hamada, *Phys. Rev. B* **2014**, *89*, 121103.
[6] M. Otani, O. Sugino, *Phys. Rev. B* **2006**, *73*, 115407.
[7] I. Hamada, M. Otani, O. Sugino, Y. Morikawa, *Phys. Rev. B* **2009**, *80*, 165411.
[8] M. J. Frisch, G. W. Trucks, H. B. Schlegel, G. E. Scuseria, M. A. Robb, J. R. Cheeseman, G. Scalmani, V. Barone, G. A. Petersson, H. Nakatsuji, X. Li, M. Caricato, A. V. Marenich, J. Bloino, B. G. Janesko, R. Gomperts, B. Mennucci, H. P. Hratchian, J. V. Ortiz, A. F. Izmaylov, J. L. Sonnenberg, D. Williams-Young, F. Ding, F. Lipparini, F. Egidi, J. Goings, B. Peng, A. Petrone, T. Henderson, D. Ranasinghe, V. G. Zakrzewski, J. Gao, N. Rega, G. Zheng, W. Liang, M. Hada, M. Ehara, K. Toyota, R. Fukuda, J. Hasegawa, M. Ishida, T. Nakajima, Y. Honda, O. Kitao, H. Nakai, T. Vreven, K. Throssell, J. A. Montgomery, Jr., J. E. Peralta, F. Ogliaro, M. J. Bearpark, J. J. Heyd, E. N. Brothers, K. N. Kudin, V. N. Staroverov, T. A. Keith, R. Kobayashi, J. Normand, K. Raghavachari, A. P. Rendell, J. C. Burant, S. S. Iyengar, J. Tomasi, M. Cossi, J. M. Millam, M. Klene, C. Adamo, R. Cammi, J. W. Ochterski, R. L. Martin, K. Morokuma, O. Farkas, J. B. Foresman, D. J. Fox, Gaussian 16 Revision C.01 **2016**, gaussian Inc. Wallingford CT.
[9] R.-P. Wang, C.-R. Hu, Y. Han, B. Yang, G. Chen, Y. Zhang, Y. Zhang, Z.-C. Dong, *J. Phys. Chem. C* **2022**, *126*, 12121.
[10] R. D. Johnson, et al., NIST Computational Chemistry Comparison and Benchmark Database **2022**, http://cccbdb.nist.gov/, NIST Standard Reference Database Number 101.
[11] B. Cirera, S. Liu, Y. Park, I. Hamada, M. Wolf, A. Shiotari, T. Kumagai, *Phys. Chem. Chem. Phys.* **2024**, *26*, 21325.
[12] M. Sun, Z. Zhang, L. Chen, S. Sheng, H. Xu, *Adv. Opt. Mater.* **2014**, *2*, 74.
[13] R. B. Jaculbia, H. Imada, K. Miwa, T. Iwasa, M. Takenaka, B. Yang, E. Kazuma, N. Hayazawa, T. Taketsugu, Y. Kim, *Nat. Nanotechnol.* **2020**, *15*, 105.
[14] D. A. Long, *The Raman Effect*, chapter 3, pages 31–48, John Wiley & Sons, Ltd. **2002**.
[15] W. A. P. Luck, T. Wess, *J. Mol. Struct.* **1992**, *270*, 229.
[16] M. Rospenk, T. Zeegers-Huyskens, *J. Phys. Chem. A* **1997**, *101*, 8428.
[17] B. Dereka, Q. Yu, N. H. Lewis, W. B. Carpenter, J. M. Bowman, A. Tokmakoff, *Science* **2021**, *371*, 160.
[18] J. Lee, K. T. Crampton, N. Tallarida, V. A. Apkarian, *Nature* **2019**, *568*, 78.
